# Low muscle mass in interstitial lung disease: a systematic review and meta-analysis of prevalence and clinical associations

**DOI:** 10.1186/s12890-026-04255-6

**Published:** 2026-04-10

**Authors:** Osamah Alsuhimi, Abdullah Alharbi, Wahaj Alnefaie, Husam Alahmadi, Fatma Almaghlouth, Abdullah A. Alqarni, Daniel Davies, Thomas M. Gill, Jean Woo, Carly Welch, Richard Beatson, Martin Kolb, John R. Hurst, Joanna C. Porter

**Affiliations:** 1 UCL Respiratory, University College London, London, UK; 2 Department of Respiratory Therapy, Faculty of Medical Rehabilitation Sciences, King Abdulaziz University, Jeddah, Saudi Arabia; 3 Yale School of Medicine, New Haven, Connecticut United States; 4 Department of Medicine, McMaster University, Hamilton, Canada; 5 Department of Medicine and Therapeutics, The Chinese University of Hong Kong CUHK University, Hong Kong, China; 6 Experimental Medicine, King’s College London, London, UK; 7 School of Cancer & Pharmaceutical Sciences, King’s College London, London, UK; 8 King Abdul Aziz University Hospital, Jeddah, Saudi Arabia; 9 University College London Hospitals NHS Foundation Trust, Rayne Institute, 114 6 University Street, London, UK

**Keywords:** Sarcopenia, Interstitial lung disease, Ageing, Pulmonary fibrosis, Mortality

## Abstract

**Background:**

Low muscle mass, a component of sarcopenia, is increasingly recognised as a marker of poor physiological reserve in chronic diseases. While observed in patients with interstitial lung disease (ILD), its prevalence and clinical associations remain inadequately characterised. Furthermore, heterogeneous diagnostic approaches from CT-derived indices to consensus definitions complicate evidence interpretation.

**Methods:**

We conducted a systematic review and meta-analysis to estimate the prevalence of low muscle mass and/or consensus-defined sarcopenia in ILD. Six databases were searched (1988–March 2024). Eight studies comprising 829 patients (701 with idiopathic pulmonary fibrosis [IPF]) met inclusion criteria. The included CT-based studies defined low muscle mass using cohort-specific lowest-quartile cut-offs, which do not meet consensus diagnostic criteria for sarcopenia.

**Results:**

The pooled prevalence of low muscle mass and/or sarcopenia was 24.3% (95% CI: 19.7–29.0) with substantial heterogeneity (I²=59.2%). A sensitivity analysis restricted to consensus-defined sarcopenia yielded a 27.5% prevalence with moderate heterogeneity (I²=62.5%); however, studies within their respective consensus frameworks (EWGSOP2 or AWGS) demonstrated zero internal heterogeneity (I²=0%). In contrast, CT-based studies using cohort-specific thresholds showed marked variability (I²=75.8%). Meta-regression confirmed diagnostic method (*p* = 0.044) and mean BMI (*p* = 0.003) as significant moderators. Low muscle mass was significantly associated with reduced pulmonary function, including lower FVC% predicted (effect size − 0.477, *p* < 0.001) and DLCO% predicted (effect size − 0.389, *p* = 0.003), as well as advancing age and lower BMI.

**Conclusions:**

While low muscle mass or consensus-defined sarcopenia affects approximately one in four ILD patients, this prevalence is largely representative of the IPF phenotype. Muscle mass abnormalities are significantly associated with respiratory decline, supporting muscle depletion as a clinically relevant marker with potential prognostic implication. However, substantial heterogeneity, driven by CT-based cohort-specific quartiles rather than externally validated thresholds, restricts the precision of current prevalence estimates. Future research should employ standardised consensus definitions, differentiate isolated muscle depletion from systemic wasting syndromes, use externally validated cut-offs, adopt multicentre prospective designs.

**Supplementary Information:**

The online version contains supplementary material available at 10.1186/s12890-026-04255-6.

## Introduction

Interstitial Lung Disease (ILD) represents a heterogeneous group of chronic, progressive respiratory disorders driven by inflammation and fibrosis of the lung interstitium. Globally, the incidence and prevalence of ILDs varies significantly, with incidence rates ranging from 1 to 31.5 per 100,000 person-years and prevalence rates from 6.3 to 71 per 100,000 individuals [1]. This variability highlights regional and diagnostic differences across studies. The mean age for ILD diagnosis calculated from multiple international studies, is 56.9 years, highlighting the mid-to-late life onset that is typical for these conditions [1]. ILD also exhibits a consistent sex disparity, with higher prevalence in men (80.9 per 100,000) compared to women (67.2 per 100,000), highlighting a greater disease burden among males. affecting predominantly older males and recognised as a prototypical disease of unhealthy ageing. A second major grouping comprises patients with ILD associated with autoimmune rheumatic diseases, who are typically, although not exclusively, younger, and middle-aged women. As global populations age, understanding how chronic diseases such as ILD intersects with systemic ageing processes, such as sarcopenia, is critical for developing equitable, person-centred approaches to chronic disease management.

Yet muscle abnormalities that may silently accelerate functional decline are often overlooked [2, 3]. Early identification of accelerated muscle loss may lead to early interventions that are essential to optimise health and offer a paradigm for management of other age-related disease [4].

Sarcopenia, defined by the European Working Group on Sarcopenia in Older People (EWGSOP) as low muscle strength accompanied by low muscle mass or quality, represents a hallmark of accelerated biological ageing that may be both a complication and a consequence of ILD [5]. Sarcopenia can be either primary (age-related) or secondary (disease-related) [6], and is increasingly being recognised as a critical factor influencing the prognosis of various chronic diseases, including ILD [7]. Importantly, modern consensus definitions require assessment of muscle function in addition to muscle mass, and imaging-based quantification of muscle size alone constitutes evidence of low muscle mass but does not fulfil criteria for sarcopenia.

Muscle dysfunction can contribute to respiratory complications by impairing respiratory muscle function, further compromising breathing capacity and overall functional status [8], while the chronic inflammation and hypoxia characteristic of ILD are hypothesised to accelerate muscle wasting through increased protein catabolism, reduced protein synthesis, and impaired muscle repair mechanisms [9, 10]. This bidirectional relationship potentially creates a cycle of declining respiratory and physical function [7]. Indeed, patients with idiopathic pulmonary fibrosis (IPF), the most common form of ILD, often demonstrate muscle dysfunction at presentation, suggesting that muscle abnormalities may be integral to the disease process rather than merely an age-related phenomenon [11–13].

Sarcopenia and cachexia represent overlapping but distinct clinical entities. Sarcopenia is defined by low muscle strength and mass, whereas cachexia is a multifactorial syndrome characterised by involuntary weight loss, systemic inflammation, and resistance to nutritional support [14]. Although both conditions manifest as muscle dysfunction, their pathophysiology and therapeutic responsiveness differ, making accurate phenotyping clinically important. In progressive fibrotic ILD, systemic inflammation and reduced appetite frequently accompany advanced disease, suggesting that a subset of patients may develop cachexia rather than sarcopenia alone; however, this distinction remains poorly characterised [15].

Recent biomarker studies have strengthened the mechanistic link between inflammation and organ dysfunction in ILD. Elevated serum levels of KL-6 and IL-18 correlate inversely with Forced vital capacity (FVC) and Diffusing Capacity of the Lungs for Carbon Monoxide (DLCO) while correlating positively with fibrotic extent on High-resolution computed tomography (HRCT), suggesting that circulating biomarkers of epithelial damage and systemic inflammation reflect both structural damage and disease activity [16]. HRCT remains essential for characterising ILD patterns and monitoring progression [17]. Whether these same inflammatory signals contribute to the muscle abnormalities observed in ILD and whether muscle status might in turn serve as a surrogate marker of systemic disease burden remains an important unanswered question.

From a clinical standpoint, muscle dysfunction in ILD may have consequences beyond functional limitation. Reduced respiratory muscle strength can compromise ventilatory reserve; diminished lean mass may limit tolerance for lung transplantation and reduce resilience during acute exacerbations; and poor exercise capacity weakens the effectiveness of pulmonary rehabilitation programmes [18]. Corticosteroid-induced myopathy, relevant to patients with inflammatory ILD subtypes, adds a further iatrogenic dimension [19]. Recognising muscle abnormalities as a potentially modifiable component of ILD morbidity could open therapeutic opportunities targeting nutrition, exercise, and pharmacological intervention.

Despite this clinical importance, the exact relationship between ILD and muscle loss remains poorly defined. Clarifying the relationship between ILD and muscle loss is essential to identify opportunities for risk stratification and to inform future intervention studies. We therefore conducted a systematic review and meta-analysis; our primary aim was to determine the prevalence of low muscle mass and/or consensus-defined sarcopenia in ILD patients, with secondary aims including examination of associations with clinical parameters and evaluation of heterogeneity introduced by different diagnostic approaches.

## Materials and methods

We conducted the review in accordance with the Cochrane Handbook for Systematic Reviews (version 6.3) and reported it according to PRISMA 2020. The protocol was registered at http://www.crd.york.ac.uk with PROSPERO (CRD42023477915). Any deviations from the protocol are listed in Supplementary Table S1).

### Search strategy

A comprehensive literature search was conducted to identify studies investigating the prevalence of sarcopenia in patients with ILD and its risk factors. Six databases, including MEDLINE (via OVID), EMBASE (via OVID), Cumulative Index to Nursing and Allied Literature (CINAHL, EBSCO host), Cochrane Central Register for Controlled Trials (CENTRAL), Scopus, and Web of Science, were searched for the term “sarcopenia” from 1988 (when the term was first used) until March 2024. Detailed search strategies, comprising a list of keywords, Medical Subject Headings (MeSH), and search strings, are available in the Supplementary Material.

### Eligibility criteria

The selected studies were exported to Rayyan [20], using Rayyan, the two authors (OA, AA) independently conducted the literature screening, reviewing titles, abstracts, and full texts based on pre-defined inclusion and exclusion criteria. Disagreements were resolved through discussion and consultation with a third reviewer (JCP). Studies were included if they:

1. Involved patients diagnosed with Interstitial Lung Disease (ILD).
2. Reported on the prevalence of sarcopenia in this population.
3. Used a clear definition and diagnostic criteria for sarcopenia.
4. Were original research articles such as cross-sectional studies, cohort studies, case-control studies, or randomised controlled trials.
5. Were published in English.

### Data extraction and quality assessment

Two authors (OA, AA) independently extracted relevant data from the included studies using a standardized form. The extracted data included study characteristics (study ID, year, country, study design, sample size), participant demographics (age, gender, ethnicity, body mass index [BMI]), diagnostic criteria for ILD and sarcopenia, ILD subtypes, and main outcomes. Disagreements between the two researchers were resolved through discussion and consultation with a third reviewer. Missing data were requested from study authors whenever possible. A prespecified modification of the Newcastle–Ottawa Scale (NOS) was applied to all included observational studies. The original NOS was developed for cohort and case–control designs and does not directly cover cross-sectional prevalence studies; therefore, the modified NOS was selected to provide a single, consistent framework across cross-sectional and cohort designs [21, 22]. Studies with NOS scores of 0–3, 4–6, and 7–9 were considered low, moderate, and high quality, respectively.

### Statistical analysis

Statistical analyses were conducted using OpenMeta[Analyst] software (Brown School of Public Health, RI) [23]. For prevalence, we used a random-effects model to calculate pooled estimates with 95% confidence intervals (CIs), reflecting between study variability. For continuous variables, effect sizes were expressed as mean differences with accompanying standard deviations (SDs). The effect sizes represent the magnitude and direction of these relationships. Mean and median with range and IQR are converted to mean, and SD based on formulas of Luo, Dehui., et al. and Wan, Xiang., et al. [24, 25]. Dichotomous outcomes were evaluated by calculating events and totals, providing a basis for effect size determination. The heterogeneity of the included studies was quantified using the I² statistic. An I² value less than 50% was considered to reflect low to moderate heterogeneity, while I² ≥ 50% indicated substantial heterogenity [26]. This threshold is commonly used in meta-analyses as it represents a moderate level of inconsistency between studies. When high heterogeneity was detected, we conducted sensitivity analysis (including exclusion of outliers) and where data permitted subgroup analysis, meta-regression, and sensitivity analysis to explore potential sources of heterogeneity and assess the robustness of the findings [27]. P-values were reported for all tests of heterogeneity to determine the presence of statistical significance, with a conventional alpha level of 0.05 used as the threshold. Data were synthesized in OpenMeta[Analyst] using random-effects models. Prevalence (proportions) was meta-analysed with binary random-effects for proportions, and continuous outcomes were pooled with continuous random-effects across studies using standardized mean differences (SMD). Pooled estimates are reported with 95% confidence intervals and I². Subgroup analyses of prevalence (pre-specified): Pooled prevalence was re-estimated within subgroups defined a priori by study design (cohort vs. cross-sectional), sample size (≥100 vs. <100), mean age (<70 vs. ≥70 years), patient group (IPF vs. mixed ILD), and sarcopenia definition (EWGSOP2, AWGS, CT-based). Each subgroup used the same random-effects model and continuity correction as the primary prevalence analysis. For each subgroup, pooled estimates with 95% CIs and heterogeneity statistics (Q, I²) are reported. Meta-regression of prevalence (exploratory): Univariable random-effects meta-regression examined mean age, mean BMI, percentage male, study design, risk-of-bias category, and sarcopenia definition as moderators. Regression coefficients (β), 95% CIs, p-values, and heterogeneity (τ²) are presented. Given the small number of studies, these analyses are considered exploratory. Associations between sarcopenia and clinical variables (secondary): Standardized mean differences (SMDs) under a random-effects model were pooled for FVC% predicted, DLCO% predicted, BMI, age, and 6-minute walk distance. SMDs with 95% CIs and heterogeneity statistics are reported. Forest plots and figures were generated for each variable and are available in the Supplementary Material.

## Results

### Identification of relevant studies

**Fig. 1:**
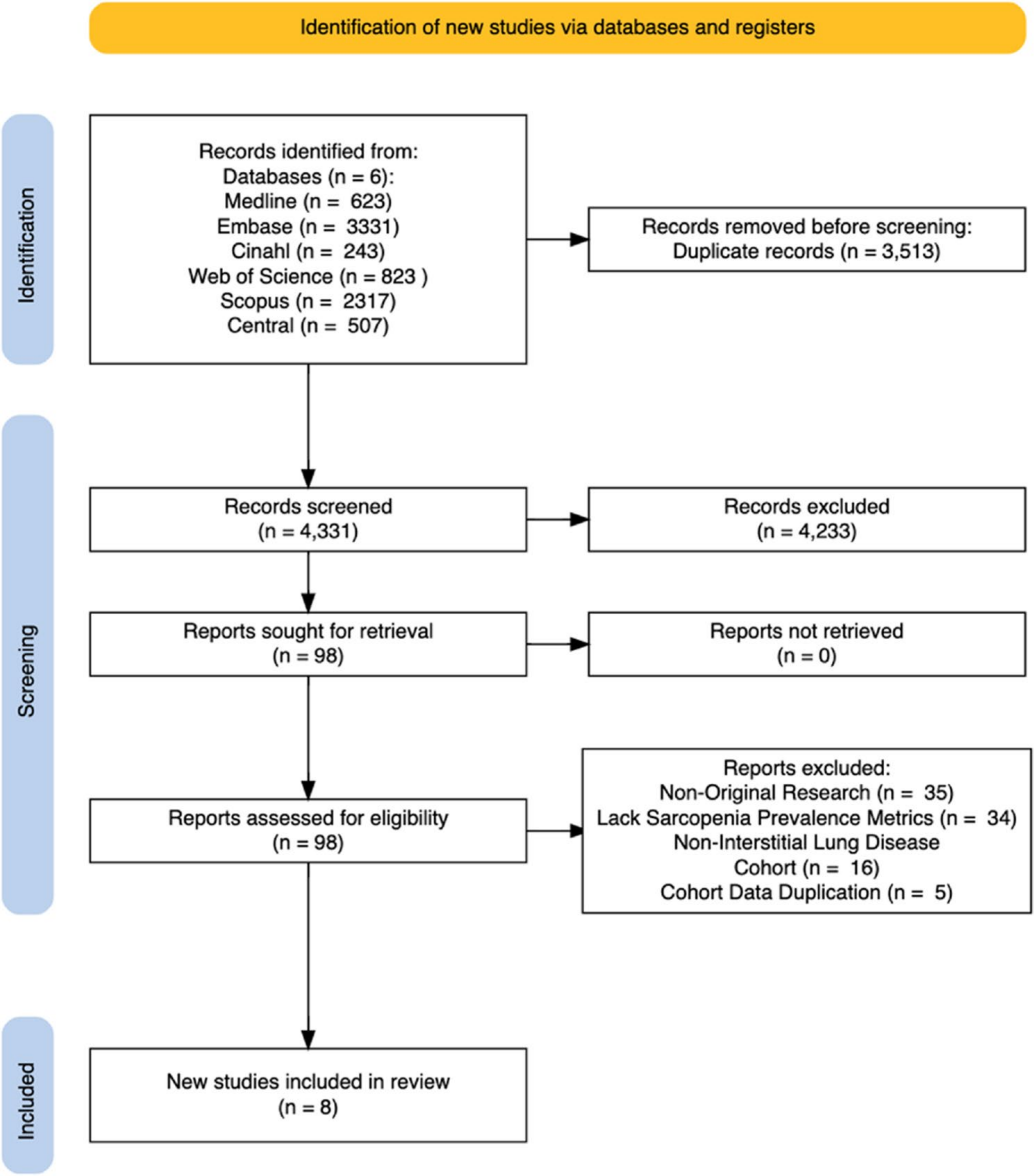
Preferred reporting items for systematic rev**ie**ws and meta-analyses (PRISMA) 2020 flow diagram [28]. A total of 8 studies fulfilled the eligibility criteria

The PRISMA flow diagram illustrating the search and selection process is shown in Figure 1. We identified a total of 7844 articles from the six databases. Following abstract screening of 4331 records, 98 full-text articles were retrieved for further screening and 90 articles were excluded, eight articles qualified for the final systematic review and meta-analysis.

### Characteristics of included studies

We included data from eight studies [29–36] with a total sample size of 829 patients with ILD, predominantly IPF (701 cases), to assess the prevalence of sarcopenia (Table 1). These studies, published between 2019 and 2024, reported a wide range of mean ages between 61.0 and 74.4 years [33]. The overall weighted mean age of participants was 70.3 years. The average sample size calculated from the table was 92 participants per study ranging from 49 to 195 participants [29–36]. The proportion of male participants ranged from 0% to 87.5%, with a weighted average of 76.8% across all studies. Table 1 outlines the characteristics of each study included in our analysis, which enrolled Japanese, Korean and Caucasian patients.

**Table 1:** Summary of included studies assessing sarcopenia in interstitial lung disease patients

Study ID	Year/ Country	Study Design	Sample Size	Diagnostic Criteria of Sarcopenia	Diagnostic Criteria of ILD	ILD Subtypes	Ethnicity	Age (Mean±SD)	Male %	BMI	Main outcomes
Çinkooğlu, A., et al. [24]	2023/Turkey	Cohort	195	Lowest Q1 of ESMA	ATS/ERS/JRS/ALAT	IPF	N/A	69.28±7.52	79	27.5± 4.07	CT-based Erector spinae muscle area (ESMA) measurements correlated with clinical markers in IPF, predicting survival.
Moon, Sung Woo., et al. [25]	2019/Korea	Cohort	180	Lowest T4MI and T12MI	ATS/ERS/JRS/ALAT	IPF	Korean	69.1(44–94*)	79.4	23.8± 3.3	Low mass at the 4th vertebra on chest CT scans significantly predicts IPF mortality.
Fujikawa, Takashi., et al. [23]	2022/Japan	Cohort	117	Lowest T4MI and T12MI	ATS/ERS/JRS/ALAT	IPF	Japanese	74.4±7.7	78.6	22.9±3.6	Carina-based sarcopenia on CT scans correlates with IPF mortality, offering a beneficial, simpler patient assessment.
Faverio, Paola., et al. [26]	2022/Italy	Cohort	83	EWGSOP2	N/A	IPF	Caucasian	72.5±6.9	80.72	27.6± 4	Sarcopenia in IPF, though not prevalent, points toward disease severity and inactivity; EWGSOP2 criteria are the efficient diagnostic method.
Hanada, Masatoshi., et al. [27]	2022/Japan	Cross-sectional	78	AWGS19	ATS/ERS/JRS/ALAT	Cryptogenic organizing pneumonia, idiopathic pulmonary fibrosis, pleuroparenchymal fibroelastosis, unclassifiable, nonspecific interstitial pneumonia, lymphoid interstitial pneumonia, combined pulmonary fibrosis and emphysema, autoimmune-ILD, Sjögren syndrome, mixed CTD-ILD, RA-ILD, hypersensitivity pneumonia.	Japanese	71.1322±2.0751	65	23.295±4.5319	CalF outperforms SARC-F and SARC-CalF in detecting sarcopenia in interstitial lung disease patients.
Sridhar, Meenakshi., et al. [30]	2024/United States	Cohort	70	Low FFMI and low handgrip Strength	ATS/ERS/JRS	IPF	N/A	70.4±6.95	68.50	29.4± 4.85	Muscle mass and sarcopenia correlated with FVC, DLCO, not 6MWD, QoL.
Fujita, Kohei., et al. [28]	2022/Japan	Cross-sectional	56	AWGS19	ATS/ERS/JRS/ALAT	IPF	Japanese	73.1±7.7	87.5	22.3± 3.1	PROs and physical performance in IPF patients are correlated with the presence of sarcopenia.
Alarcón-Dionet, A., et al. [29]	2024/Mexico	Cross-sectional	50	EWGSOP	ATS/ERS 2013	Hypersensitivity Pneumonitis Autoimmune-ILD	N/A	61±11	0	Reported as N(%)	In multivariate analysis, low appendicular muscle mass and SARC-F were correlated with malnutrition.

*Q1* First quartile, *ESMA* Erector spinae muscle area, *ATS/ERS/JRS/ALAT* collaboration among American Thoracic Society, European Respiratory Society and Japanese Respiratory Society, *CT* Computed Tomography, *T4MI and T12MI* Thoracic 4 and Thoracic 12 Muscle Index, *CalF* Calf circumference, *SARC-F* Strength, Assistance in walking, Rising from

### Sarcopenia diagnostic criteria and measurements

Analysis of the included studies revealed three distinct diagnostic approaches for sarcopenia (Table 2). Two studies employed the European Working Group on Sarcopenia in Older People (EWGSOP2) criteria [32, 35], two utilised the Asian Working Group for Sarcopenia (AWGS) criteria [33, 34], and four studies used CT-based assessments with low muscle mass defined as values falling within the lowest quartile of the study population [29, 30, 36].

**Table 2:** Sarcopenia diagnostic criteria across included studies

Diagnostic Approach	Studies	Measurement Components	Cut-off Values
EWGSOP2	2	• Muscle strength (handgrip)	Men: <27 kg
Faverio., et al. [32]		• Muscle mass	Women: <16 kg
Alarcón-Dionet., et al. [35]		• Physical performance	Gait speed: ≤0.8 m/s
AWGS	2	• Muscle strength (handgrip)	Men: <28 kg
Hanada., et al. [33]		• Muscle mass (SMI)	Women: <18 kg
Fujita, Kohei., et al. [34]			SMI men: <7.0 kg/m²
			SMI women: <5.7 kg/m²
CT-based Assessment	4	• Pectoralis muscle CSA at T4	Lowest quartile
Sridhar, Meenakshi., et al. [36]		• Erector spinae muscle at T12	by gender within
Çinkooglu., et al. [30]		• Muscle indices (area/height²)	study population
Fujikawa., et al. [29]			
Moon, Sung Woo., et al. [31]			

This table summarizes the three diagnostic approaches for sarcopenia used across included studies: EWGSOP2 criteria [24, 27] combining low handgrip strength (<27 kg men; <16 kg women), low muscle mass and gait speed ≤0.8 m/s; AWGS criteria (Hanada et al.; Fujita et al.) based on handgrip <28 kg (men), SMI <7.0 kg/m² (men) or <5.7 kg/m² (women) (female SMI <18 kg); and CT-based assessments (four studies) measuring muscle cross-sectional area at T4 or T12 or area/height², with sarcopenia defined as the lowest gender-specific quartile

Muscle mass measurements were performed using three different methods across the studies (Table 3). Bioelectrical impedance analysis (BIA) in four studies [32–35], CT imaging in four studies [29–31, 36], and dual-energy X-ray absorptiometry (DEXA) in two studies [34, 35]. CT-based measurements included pectoralis muscle cross-sectional area at the T4 vertebral level and erector spinae muscle measurements at T12, with tissue attenuation ranging from −30 to +150 Hounsfield units (HU). All studies using EWGSOP2 or AWGS criteria employed handgrip strength as the measure of muscle strength, with different cut-off values for European (male <27 kg; female <16 kg) and Asian (male <28 kg; female <18 kg) populations.

**Table 3:** Muscle mass measurement methods across included studies

Method	Studies	Parameters Assessed
BIA	4	muscle mass (via InBody S10); total body composition.
Faverio., et al. [32]		
Hanada., et al. [33]		
Fujita, Kohei., et al. [34]		
Alarcón-Dionet., et al. [35]		
CT Imaging	4	Muscle cross-sectional area; muscle attenuation (−30 to +150 HU); muscle area index (area/height²).
Sridhar, Meenakshi., et al. [36]		
Çinkooglu., et al. [30]		
Fujikawa., et al. [29]		
Moon, Sung Woo., et al. [31]		
DEXA	2	Appendicular muscle mass (whole-body DEXA); body composition
Fujita, Kohei., et al. [34]		
Alarcón-Dionet., et al. [35]		

Muscle mass in ILD was quantified by bioelectrical impedance analysis (four studies), CT imaging (four studies), and DEXA (two studies), each providing complementary data on muscle quantity, quality (attenuation), and distribution. Bioelectrical impedance was measured using the InBody S10 device, a multi-frequency segmental analyser designed for clinical use in immobile patients. These methods together map the full spectrum of body composition changes relevant to disease severity and prognosis

### Sarcopenia prevalence

**Fig. 2:**
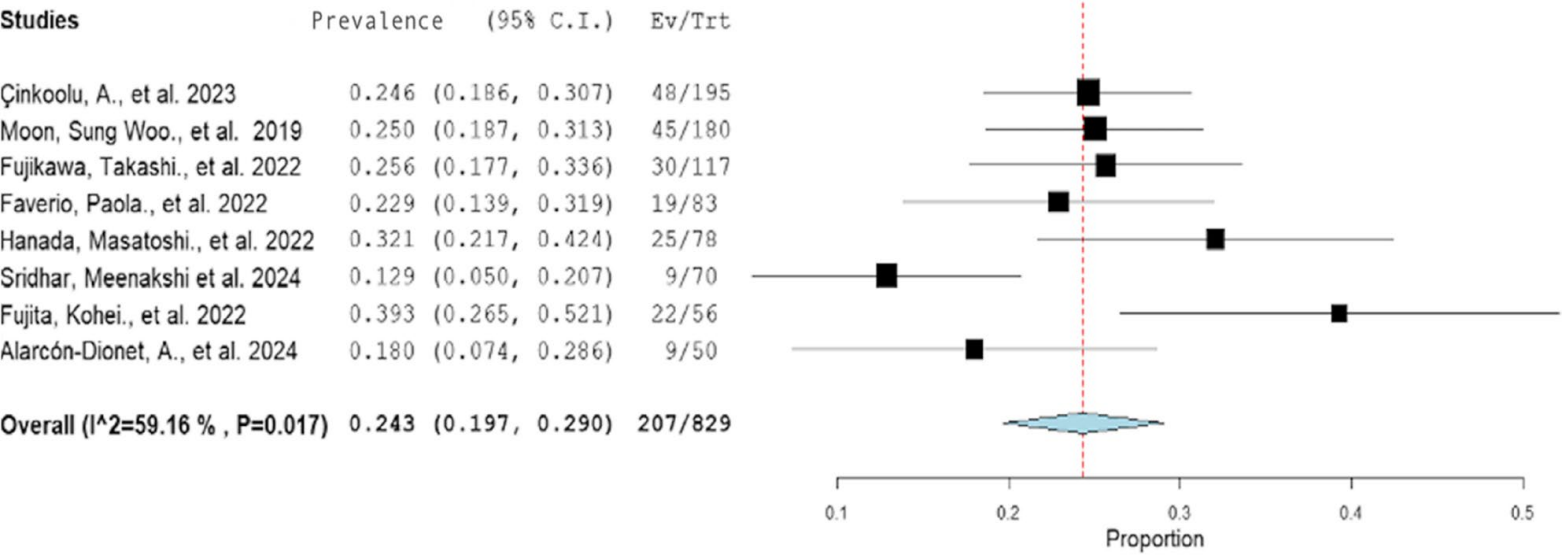
Forest plot showing the prevalence of sarcopenia among patients with interstitial lung disease. The meta-analysis included data from 829 patients in 8 studies. Each black square represents an individual study’s estimated prevalence (Ev/Trt) and its 95% confidence interval (horizontal line); square size represents the study weight. The blue diamond shows the pooled prevalence (0.243) and its 95% CI (0.197–0.290.197.290). the red dashed vertical line marks that overall estimate. Studies with squared left of the line reported below-average prevalence; those to the right were above average

The meta-analysis revealed a pooled prevalence of sarcopenia of 24.3% (95% CI: 19.7%–29.0%) among patients with ILD, with high heterogeneity (I^2^ = 59.2%, *p* = 0.017; Figure 2). The studies by Fujita, Kohei., et al. and Sridhar, Meenakshi., et al. reported the lowest and highest sarcopenia prevalence respectively, and these outliers affected the overall results. Excluding these studies reduced the heterogeneity (I^2^ = 0%, *p* = 0.598) and slightly adjusted the pooled prevalence to 24.8% (95% CI: 21.6%, 28%), data not shown, however all 8 were retained for the remainder of the analyses.

### Subgroup analyses of sarcopenia prevalence

The prevalence and associated confidence intervals of sarcopenia when splitting the pooled populations into individual subcategories: study design, sample size, study quality, age, patient groups, and sarcopenia measurement methods are demonstrated in Table 4. In terms of study design, cohort studies [29–32] indicated a prevalence of 24.7% (95% CI: 21.2%−28.2%), and cross-sectional studies [33–36] reported a 25% prevalence (95% CI: 13%−36.9%). Sample sizes greater than 100^29–31^ show a prevalence of 25% (95% CI: 21.2%−28.8%), while those with fewer than 100^32–36^ have a prevalence of 24.4% (95% CI: 15.4%−33.4%). High-quality studies [30–33] have a prevalence of 25.4% (95% CI: 21.7%−29.1%), and medium-quality studies [29], [34–36] show 23.2% (95% CI: 13.1%−33.4%). Age-based subgroup analysis revealed a sarcopenia prevalence of 23.8% (95% CI: 19.8%–27.9%) in studies with a mean age under 70 years, and 24.3% (95% CI: 14.8%–33.8%) in those over 70 years. For patient groups, ILD [31, 35] was associated with a prevalence of 22.9% (95% CI: 16.5%−29.2%), and in those studies that considered IPF alone [29, 30], [32–34, 36] there was a prevalence of 25.3% (95% CI: 19%−31.6%). Sarcopenia measurement methods yield different prevalence: CT scans [29–31, 36] show 22.3% (95% CI: 16.8%−27.8%), EWGSOP2^32, 35^ criteria indicate 20.8% (95% CI: 14%−27.7%), and AWGS [33, 34] criteria report a prevalence of 34.6% (95% CI: 26.9%−43%). Given this variance across diagnostic tools, a sensitivity analysis excluding CT-based studies and restricted exclusively to consensus-defined sarcopenia (EWGSOP2 or AWGS criteria) was performed. This analysis yielded a pooled prevalence of 27.5% (95% CI: 18.9%–36.2%) across four studies comprising 267 participants.

**Table 4:** Overview of the prevalence of sarcopenia across various subcategories

Category	No. of Studies	Prevalence (95% CI)	Heterogeneity test	
			*P*	I^2^
Study Design				
Cohort	4	24.7% (21.2% to 28.2%)	0.975	0%
Cross-sectional	4	25% (13% to 36.9%)	0.001*	81.7%
Sample Size				
>100	3	25% (21.2% to 28.8%)	0.980	0%
<100	5	24.4% (15.4% to 33.4%)	0.003*	75.5%
Study Quality				
High	4	25.4% (21.7% to 29.1%)	0.581	0%
Medium	4	23.2% (13.1% to 33.4%)	0.004*	77.8%
Age				
<70 years	3	23.8% (19.8% to 27.9%	0.510	0%
>70 years	4***	24.3% (14.8% to 33.8%)	0.005*	76.99%
Patient Groups				
ILD	2	22.9% (16.5%, 29.2%)	0.268	18.5%
IPF	6	25.3% (19%−31.6%)	0.007*	68.4%
Sarcopenia Measure				
CT	4	22.3% (16.8%- 27.8%)	0.058	59.8%
EWGSOP	2	20.8% (14%, 27.7%)	0.492	0%
AWGS	2	34.6% (26.9%−43%)	0.389	0%
Consensus-defined sarcopenia (EWGSOP2 and AWGS criteria)	4	27.5% (18.9%–36.2%)	0.046*	62.5%

*, Presence of statistical significance, with a conventional alpha level of 0.05 used as the threshold

***, Hanada, Masatoshi., et al. excluded from the age subgroup analysis because they reported the median instead of mean

### Meta-regression analysis

There was considerable variability in the prevalence of sarcopenia among individuals with ILD, as indicated by high heterogeneity (I^2^ = 59.16%, *P* = 0.017). To further explore variables that might influence the prevalence of sarcopenia in ILD, a meta-regression was conducted. The analysis, detailed in Table 5, shows six variables: study design, study quality, mean age, mean BMI, percentage male, and sarcopenia measurement criteria. The analysis revealed that mean BMI showed a weak but significant negative association (*r* = –0.018), indicating that as BMI increased, the prevalence of sarcopenia decreased, while the choice of sarcopenia definition was positively associated with prevalence (*r* = 0.050). All other factors (design, quality, age, sex ratio) showed no effect.

**Table 5:** Meta-regression analysis of potential variables affecting the prevalence of sarcopenia in ILD

Moderator	Regression coefficient	95% CI	*P* value
Study design	−0.009	−0.096 to 0.078	0.845
Study quality	−0.034	−0.114 to 0.046	0.406
Mean age	0.008	−0.004 to 0.019	0.192
Mean BMI	−0.018	−0.030 to −0.006	0.003*
Male percentage	0.004	−0.002 to 0.011	0.179
Sarcopenia measure	0.050	0.001 to 0.098	0.044*

Six study-level factors were tested for their effect on between-study heterogeneity (I² = 59.2%, *p* = 0.017). Mean BMI (β = –0.018, 95% CI –0.030 to –0.006; *p* = 0.003) and sarcopenia definition (β = 0.050, 95% CI 0.001–0.098; *p* = 0.044) were the only significant moderators. The negative BMI coefficient means each one-unit rise in average BMI corresponded to a 1.8-point drop in sarcopenia prevalence (CI: 0.6–3.0 points). The positive definition coefficient means switching criteria raised prevalence estimates by 5 points (CI: 0.1–9.8 points). All other factors (design, quality, age, sex ratio) showed no effect

### Analysis of factors associated with sarcopenia in patients with ILD

**Table 6:** Effect sizes and statistical significance of factors associated with sarcopenia prevalence in ILD patients

Factor	Effect Size	95% CI (Lower, Upper)	Std. Error	*P*-Value (Effect)
FVC%	−0.477	(−0.747, −0.207)	0.138	<0.001
FVC% Predicted	−0.357	(−0.594, −0.120)	0.121	0.003
DLCO% Predicted	−0.389	(−0.649, −0.128)	0.133	0.003
Age	0.328	(0.103, 0.554)	0.115	0.004
BMI	−0.394	(−0.726, −0.062)	0.169	0.020
6MWT	−0.710	(−1.559, 0.140)	0.433	0.102
Smoking Pack-Year	−0.139	(−0.330, 0.052)	0.097	0.154
Male%	1.002	(0.671, 1.333)	-	0.991

For each factor, the table reports the standardized difference in sarcopenia prevalence (effect size), its 95% confidence interval, standard error, and p-value. Negative effect sizes indicate that sarcopenic patients had lower values than non-sarcopenic patients (e.g. FVC% was 0.477 SD lower, 95% CI –0.747 to –0.207, *p*<0.001), whereas positive effect sizes indicate higher values (e.g. age was 0.328 SD higher per year, 95% CI 0.103–0.554, *p*=0.004). Factors with *p*<0.05 (FVC%, DLCO%, age, BMI) are significantly associated with sarcopenia prevalence; 6MWT, smoking history, and male% were not

The number of contributing studies per outcome was small, and heterogeneity varied across analyses. The results from a meta-analysis investigating factors associated with sarcopenia in patients with ILD are presented in Table 6. Standardised mean differences (SMD) between ILD patients with and without sarcopenia were quantified for selected clinical parameters. In our analysis of pulmonary function, FVC% demonstrated a moderate negative relationship (effect size −0.477, *p*<0.001), indicating that lower FVC% values are associated with higher sarcopenia prevalence. Similarly, ‘FVC% predicted’ and ‘DLCO% predicted’ showed moderate negative relationships (effect sizes −0.357 and −0.389 respectively, both *p*=0.003), showing that decreased pulmonary function correlates with increased sarcopenia prevalence. Regarding physical parameters, BMI showed a moderate negative relationship (effect size −0.394, *p*=0.020), indicating that lower BMI values are associated with higher sarcopenia prevalence. Age demonstrated a moderate positive relationship (effect size 0.328, *p*=0.004), suggesting that increased age is associated with higher sarcopenia prevalence. The 6-minute walk test (6MWT) showed a strong but non-significant negative relationship (effect size −0.710, *p*=0.102). Each effect size calculation included studies with varying sample sizes, and detailed forest plots for each parameter are available in the supplementary materials.

## Discussion

Sarcopenia and low muscle mass play an important role in modulating lung function in ILD patients [32]. This systematic review is the first to our knowledge to synthesise sarcopenia and low muscle mass prevalence estimates across all ILD subtypes and thus broadens previous IPF-only analyses [37]. Our findings identify muscle abnormalities as a clinically significant comorbidity in ILD; this is particularly relevant given that sarcopenia was formally recognised as a disease in 2016 [38].

The heterogeneity in diagnostic approaches observed in our analysis reflects a broader challenge in sarcopenia research, particularly in the context of secondary sarcopenia associated with chronic diseases. In contrast to primary sarcopenia, which affects approximately 5–13% [39] of individuals aged 60–70 years, our pooled prevalence of sarcopenia 24.3% indicates a substantially higher burden in ILD populations. The precision of the pooled data remains compromised, as cohort-specific quartile thresholds inflate prevalence estimates compared to external cut-offs.

The three diagnostic approaches identified in this review differ fundamentally in their conceptual basis and clinical application. EWGSOP2, developed primarily from European populations, employs a stepwise algorithm requiring demonstration of low muscle strength (handgrip <27 kg for men, <16 kg for women) as the primary criterion, with low muscle mass serving as confirmation. AWGS applies similar principles but uses thresholds calibrated to Asian body composition (handgrip <28 kg for men, <18 kg for women) and permits either dual-energy X-ray absorptiometry or bioelectrical impedance for mass quantification (Table 2) [40]. Importantly, both frameworks require functional assessment, ensuring that identified patients have demonstrable impairment rather than isolated anatomical findings. This provides a level of functional specificity absent in CT-only assessments, where mass quantification serves as a surrogate for the clinical syndrome but omits the requisite functional evaluation mandated by international consensus.

In our review, four studies quantified muscle cross-sectional area at standardised vertebral levels (T4 or T12) using CT imaging. These studies defined low muscle mass as values falling within the lowest sex-specific quartile of each study population (Table 3). This methodological approach presents two critical interpretive challenges. First, by defining low muscle mass statistically rather than pathologically, this method artificially fixes the prevalence at approximately 25%. Such a constraint can bias our pooled estimate toward one in four and conflates relative muscle depletion within a specific cohort with the distinct biological construct of clinical sarcopenia. Second, because these quartile-based definitions merely reflect the statistical distribution within a given cohort, they do not establish true pathological thresholds. The clinical impact of this flaw is apparent in advanced IPF. Given that these patients frequently present with a low baseline BMI, even individuals falling into a statistically ‘normal’ quartile may already be pathologically depleted compared to external reference groups. Therefore, the reliance on cohort-specific, quartile-based definitions precludes any inference regarding the absolute prevalence of sarcopenia in ILD relative to population norms.

The decision to pool studies using different assessment methods requires careful methodological consideration. As CT-based studies merely assess relative low muscle mass, we conducted a sensitivity analysis restricted exclusively to studies utilising formal consensus-defined sarcopenia criteria (EWGSOP2 and AWGS). This restricted analysis yielded a pooled prevalence of 27.5%. Nevertheless, this subgroup still demonstrated moderate heterogeneity (I² = 62.5%). However, a closer examination reveals that this variance is driven by methodological differences in population-specific thresholds. The divergence between European (EWGSOP2: 20.8%) and Asian (AWGS: 34.6%) prevalence estimates reflects their distinct, built-in diagnostic cut-offs for muscle mass and strength. Studies using consensus-based definitions demonstrated zero internal heterogeneity when analysed within their respective frameworks. In complete contrast, CT-based studies using cohort-specific quartiles showed marked and persistent variability (I²=75.8%).

These observations align with our meta-regression analysis, which identified diagnostic method and mean BMI as significant moderators of prevalence. The positive association between diagnostic definition and prevalence reflects this methodological heterogeneity rather than true biological variability. Consequently, these findings identify study-level trends rather than individual-level clinical associations.

At the biological level, the mechanisms driving sarcopenia in ILD involve chronic inflammation and oxidative stress [41–44]. These pathways exacerbate muscle degradation across all ILD subgroups, but are particularly relevant in IPF, the most common and progressive form of the disease, typically affecting patients over 60 years of age [45]. Inflammatory cytokines such as tumour necrosis factor-alpha (TNF-α) and interleukin-6 (IL-6) are often elevated in ILD, promoting protein breakdown over synthesis and leading to muscle wasting [46]. Moreover, the physical inactivity resulting from impaired lung function and dyspnoea exacerbates muscle loss, creating a cycle that further diminishes patient quality of life [13, 47, 48]. Consistent with this functional decline, we found low muscle mass significantly correlates with reduced FVC and DLCO. Despite these clinically relevant effect sizes, the limited number of studies tracking these specific parameters, alongside their variable heterogeneity, necessitates cautious interpretation. While the standardized mean difference indicated a considerable negative association with 6MWT, this finding remained statistically non-significant. This discrepancy is likely driven by limited study power and the multifactorial nature of exercise limitation in ILD, where muscle depletion further compounds an already impaired ventilatory reserve.

The current literature does not allow for a reliable clinical distinction between secondary sarcopenia and cachexia in ILD. Cachexia, characterised by involuntary weight loss, systemic inflammation, and anorexia unresponsive to nutritional support, has recently been reported in 25–42% of IPF patients [15]. Because advanced fibrotic ILD is frequently accompanied by systemic inflammation and weight loss, making phenotype misclassification probable. Both sarcopenia and cachexia are associated with reduced body weight, making BMI a practical initial screening marker in ILD. A consideration specific to ILD is the potential impact of disease-modifying therapies on muscle status. The antifibrotics, nintedanib and pirfenidone, although reducing the rate of progressive fibrosis, are associated with appetite reduction and weight loss and themselves may have an impact on sarcopenia.

This study’s major strength lies in its comprehensive approach across a broad spectrum of ILD patients; however, several limitations should be acknowledged. The main limitation of this review is definitional heterogeneity, which results in a pooled prevalence that reflects a composite of muscle phenotypes rather than an absolute measure of clinical sarcopenia. Even when formal consensus frameworks (EWGSOP2 and AWGS) are applied, their diagnostic thresholds reflect validations in community-dwelling older adults; as a result, physical performance metrics in ILD may be confounded by exertional dyspnoea or hypoxia, reflecting ventilatory limitation rather than true muscle dysfunction. Over 80% of the pooled patients were diagnosed with IPF. The findings reflect that specific population and tend to misrepresent the biological reality of other ILD subtypes. Alongside cohort imbalance, the overall evidence base remains constrained to eight studies comprising fewer than 1000 patients. Findings derived from single-centre observational data constrain the generalisability of the results. This is further influenced by the geographic distribution of the evidence, where the absence of large, multicentre European or North American registry-based studies prevents a comprehensive assessment of sarcopenia across diverse global populations. Finally, the lack of formal cachexia diagnostic criteria across the included primary literature prevents the accurate differentiation of sarcopenia from overlapping inflammatory wasting syndromes.

These observations support specific methodological recommendations for future research. Studies should employ consensus-based definitions (EWGSOP2 or AWGS, selected appropriately for the study population) incorporating both muscle mass and function, enabling cross-study comparison and identification of patients with demonstrable functional impairment. When CT-based assessment is used for opportunistic screening during routine ILD monitoring, externally validated thresholds derived from reference populations should replace cohort-specific quartiles to permit meaningful prevalence estimation. Prospective designs should incorporate longitudinal weight trajectories, inflammatory biomarkers, and formal cachexia diagnostic criteria to isolate secondary sarcopenia from systemic wasting syndromes. Protocols should report sufficient methodological detail regarding measurement protocols, cut-off derivation, and population characteristics to facilitate appropriate interpretation and future meta-analysis. Standardisation of diagnostic criteria across ILD sarcopenia research is essential to improve comparability and clinical applicability of findings.

Translating the current evidence into clinical practice broadens healthcare strategies for patients with ILD. This meta-analysis identifies sarcopenia and low muscle mass as prevalent and potentially modifiable risk factors in ILD, and an increasingly recognised component of the multimorbidity burden in lung fibrosis. Several strategies merit consideration for integrating muscle assessment into routine ILD care. First, opportunistic screening using CT imaging obtained for routine ILD monitoring can identify patients with anatomical muscle depletion, which should ideally trigger subsequent functional assessment. Second, simple functional tests including handgrip dynamometry can be incorporated into outpatient clinic visits with minimal time and resource requirements. Third, patients identified with muscle abnormalities should be evaluated for modifiable contributors including nutritional deficiency, physical inactivity, and medication effects. Embedding routine muscle screening into ILD care pathways offers a cost-effective, scalable opportunity to improve outcomes in ILD patients [49]. Recognising sarcopenia and low muscle mass as modifiable risk factors with prognostic significance highlights the need for integrated muscle assessment in ILD care, ideally incorporating both mass quantification and functional evaluation as recommended by consensus guidelines.

## Supplementary Information

Supplementary Material 1.

## Data Availability

As this is a systematic review, all data are already available in published articles. All data analysed for this review are summarized in the supplementary material.

## Abbreviations

6MWT: six-minute walk test
AWGS: Asian Working Group for Sarcopenia
BIA: Bioelectrical Impedance Analysis
BMI: body mass index
CENTRAL: Cockrane Register for Controlled Trials
CI: Confidence Interval
CINAHL: Cumulative Index to Nursing and Allied Literature
CSA: Cross Sectional Area
CT: Computed Tomography
DEXA: Dual-energy X-ray absorptiometry
DLCO: Diffusing Capacity of the Lungs for Carbon Monoxide
EWGSOP2: European Working Group on Sarcopenia in Older People
FVC: Forced vital capacity
GAP: Gender-age-physiology
HADS: Hospital Anxiety and Depression Scale
IL-6: Interleukin 6
ILD: Interstitial lung disease
IPF: Idiopathic pulmonary fibrosis
Kg: Kilogram
MeSH: Medical Subject Headings
MRC: Medical research council
NOS: Newcastle-Ottawa Scale
PRISMA: Preferred Reporting Items for Systematic Review and Meta-analyses
SARQ: St. George’s Respiratory Questionnaire
SARC-F: Strength, Assistance with walking, Rise from a chair, Climb stairs, and Falls
SMD: Standardised mean difference
SMI: Muscle index
T4: Thoracic spine vertebrae 4
T12: Thoracic spine vertebrae 12
TNF-α: Tumour necrosis factor alpha
