# Supplementary material for: Low muscle mass in interstitial lung disease: a systematic review and meta-analysis of prevalence and clinical associations
Source: BMC Pulm Med. 2026 Apr 10;26:229. doi: 10.1186/s12890-026-04255-6 (PMC13188284; doi:10.1186/s12890-026-04255-6)
Supplement: Supplementary file 1 — Supplementary Material 1. [file 12890_2026_4255_MOESM1_ESM.docx]

1. **The search strategy:**

**Association + Prevalence: Search Strategy:**

CINAHL Plus

Search Alert: "TX (("interstitial lung disease*" OR "Diffuse Parenchymal Lung Disease*" OR "Lung Fibrosis" OR "Granulomatous Disease*" OR "Alveolitis" OR "Interstitial pneumonia" OR "Nonspecific interstitial pneumonitis" OR "sarcoidosis" OR "hypersensitivity pneumonitis" OR "Cryptogenic alveolitis" OR "fibrosing alveolitis" OR "Idiopathic pulmonary fibrosis" OR "asbestosis" OR "Silicosis" OR "Berylliosis" OR "Bagassosis" OR "Pneumoconiosis" OR "Vasculitis" OR "Hamman-Rich Syndrome" OR "Histiocytosis X" OR "Lymphangioleiomyomatosis" OR "Langerhans Cell Histiocytosis" OR "Desquamative interstitial pneumonia" OR "Acute interstitial pneumonia" OR "bronchiolitis obliterans organizing pneumonia" OR "Cryptogenic organizing pneumonia" OR "Lymphoid interstitial pneumonia") AND ("sarcopenia" OR "muscle mass" OR "muscle quantity" OR "muscle strength" OR "muscle quality" OR "physical performance" OR "muscle wasting" OR "muscle atrophy" OR "muscle function" OR "muscle performance" OR "muscle loss")) Publication Year: 1988-2023 AND Also search within the full text of the articles on 2024-01-31 04:57 PM"

Cochrane Library:

(("Interstitial Lung Disease" OR "Diffuse Parenchymal Lung Disease" OR "Lung Fibrosis" OR "Granulomatous Disease" OR "Alveolitis" OR "Interstitial pneumonia" OR "Nonspecific interstitial pneumonitis" OR "sarcoidosis" OR "hypersensitivity pneumonitis" OR "Cryptogenic alveolitis" OR "fibrosing alveolitis" OR "Idiopathic pulmonary fibrosis" OR "asbestosis" OR "Silicosis" OR "Berylliosis" OR "Bagassosis" OR "Pneumoconiosis" OR "Vasculitis" OR "Hamman-Rich Syndrome" OR "Histiocytosis X" OR "Lymphangioleiomyomatosis" OR "Langerhans Cell Histiocytosis" OR "Desquamative interstitial pneumonia" OR "Acute interstitial pneumonia" OR "bronchiolitis obliterans organizing pneumonia" OR "Cryptogenic organizing pneumonia" OR "Lymphoid interstitial pneumonia") AND ("sarcopenia" OR "muscle mass" OR "muscle quantity" OR "muscle strength" OR "muscle quality" OR "physical performance" OR "muscle wasting" OR "muscle atrophy" OR "muscle function" OR "muscle performance" OR "muscle loss")) in **Title Abstract Keyword**, with Cochrane Library publication date from Jan 1988 to Nov 2023, (Word variations have been searched).

EMBASE:

(("interstitial lung disease*" or "Diffuse Parenchymal Lung Disease*" or "Lung Fibrosis" or "Granulomatous Disease*" or "Alveolitis" or "Interstitial pneumonia" or "Nonspecific interstitial pneumonitis" or "sarcoidosis" or "hypersensitivity pneumonitis" or "Cryptogenic alveolitis" or "fibrosing alveolitis" or "Idiopathic pulmonary fibrosis" or "asbestosis" or "Silicosis" or "Berylliosis" or "Bagassosis" or "Pneumoconiosis" or "Vasculitis" or "Hamman-Rich Syndrome" or "Histiocytosis X" or "Lymphangioleiomyomatosis" or "Langerhans Cell Histiocytosis" or "Desquamative interstitial pneumonia" or "Acute interstitial pneumonia" or "bronchiolitis obliterans organizing pneumonia" or "Cryptogenic organizing pneumonia" or "Lymphoid interstitial pneumonia") and ("sarcopenia" or "muscle mass" or "muscle quantity" or "muscle strength" or "muscle quality" or "physical performance" or "muscle wasting" or "muscle atrophy" or "muscle function" or "muscle performance" or "muscle loss")).mp. limit 1 to yr="1988 -Current"

MedLine:

(("interstitial lung disease*" or "Diffuse Parenchymal Lung Disease*" or "Lung Fibrosis" or "Granulomatous Disease*" or "Alveolitis" or "Interstitial pneumonia" or "Nonspecific interstitial pneumonitis" or "sarcoidosis" or "hypersensitivity pneumonitis" or "Cryptogenic alveolitis" or "fibrosing alveolitis" or "Idiopathic pulmonary fibrosis" or "asbestosis" or "Silicosis" or "Berylliosis" or "Bagassosis" or "Pneumoconiosis" or "Vasculitis" or "Hamman-Rich Syndrome" or "Histiocytosis X" or "Lymphangioleiomyomatosis" or "Langerhans Cell Histiocytosis" or "Desquamative interstitial pneumonia" or "Acute interstitial pneumonia" or "bronchiolitis obliterans organizing pneumonia" or "Cryptogenic organizing pneumonia" or "Lymphoid interstitial pneumonia") and ("sarcopenia" or "muscle mass" or "muscle quantity" or "muscle strength" or "muscle quality" or "physical performance" or "muscle wasting" or "muscle atrophy" or "muscle function" or "muscle performance" or "muscle loss")).mp.

2. limit 1 to yr="1988 -Current"

Web of Science

ALL=(("interstitial lung disease*" OR "Diffuse Parenchymal Lung Disease*" OR "Lung Fibrosis" OR "Granulomatous Disease*" OR "Alveolitis" OR "Interstitial pneumonia" OR "Nonspecific interstitial pneumonitis" OR "sarcoidosis" OR "hypersensitivity pneumonitis" OR "Cryptogenic alveolitis" OR "fibrosing alveolitis" OR "Idiopathic pulmonary fibrosis" OR "asbestosis" OR "Silicosis" OR "Berylliosis" OR "Bagassosis" OR "Pneumoconiosis" OR "Vasculitis" OR "Hamman-Rich Syndrome" OR "Histiocytosis X" OR "Lymphangioleiomyomatosis" OR "Langerhans Cell Histiocytosis" OR "Desquamative interstitial pneumonia" OR "Acute interstitial pneumonia" OR "bronchiolitis obliterans organizing pneumonia" OR "Cryptogenic organizing pneumonia" OR "Lymphoid interstitial pneumonia") AND ("sarcopenia" OR "muscle mass" OR "muscle quantity" OR "muscle strength" OR "muscle quality" OR "physical performance" OR "muscle wasting" OR "muscle atrophy" OR "muscle function" OR "muscle performance" OR "muscle loss")) Publication Date 1988-01-01 to 2023-11-21.

Scopus:

TITLE-ABS-KEY(( ( "interstitial lung disease*" OR "Diffuse Parenchymal Lung Disease*" OR "Lung Fibrosis" OR "Granulomatous Disease*" OR "Alveolitis" OR "Interstitial pneumonia" OR "Nonspecific interstitial pneumonitis" OR "sarcoidosis" OR "hypersensitivity pneumonitis" OR "Cryptogenic alveolitis" OR "fibrosing alveolitis" OR "Idiopathic pulmonary fibrosis" OR "asbestosis" OR "Silicosis" OR "Berylliosis" OR "Bagassosis" OR "Pneumoconiosis" OR "Vasculitis" OR "Hamman-Rich Syndrome" OR "Histiocytosis X" OR "Lymphangioleiomyomatosis" OR "Langerhans Cell Histiocytosis" OR "Desquamative interstitial pneumonia" OR "Acute interstitial pneumonia" OR "bronchiolitis obliterans organizing pneumonia" OR "Cryptogenic organizing pneumonia" OR "Lymphoid interstitial pneumonia" ) AND ( "sarcopenia" OR "muscle mass" OR "muscle quantity" OR "muscle strength" OR "muscle quality" OR "physical performance" OR "muscle wasting" OR "muscle atrophy" OR "muscle function" OR "muscle performance" OR "muscle loss" ) )) AND PUBYEAR > 1986 AND PUBYEAR < 2024.

Regular prevalence


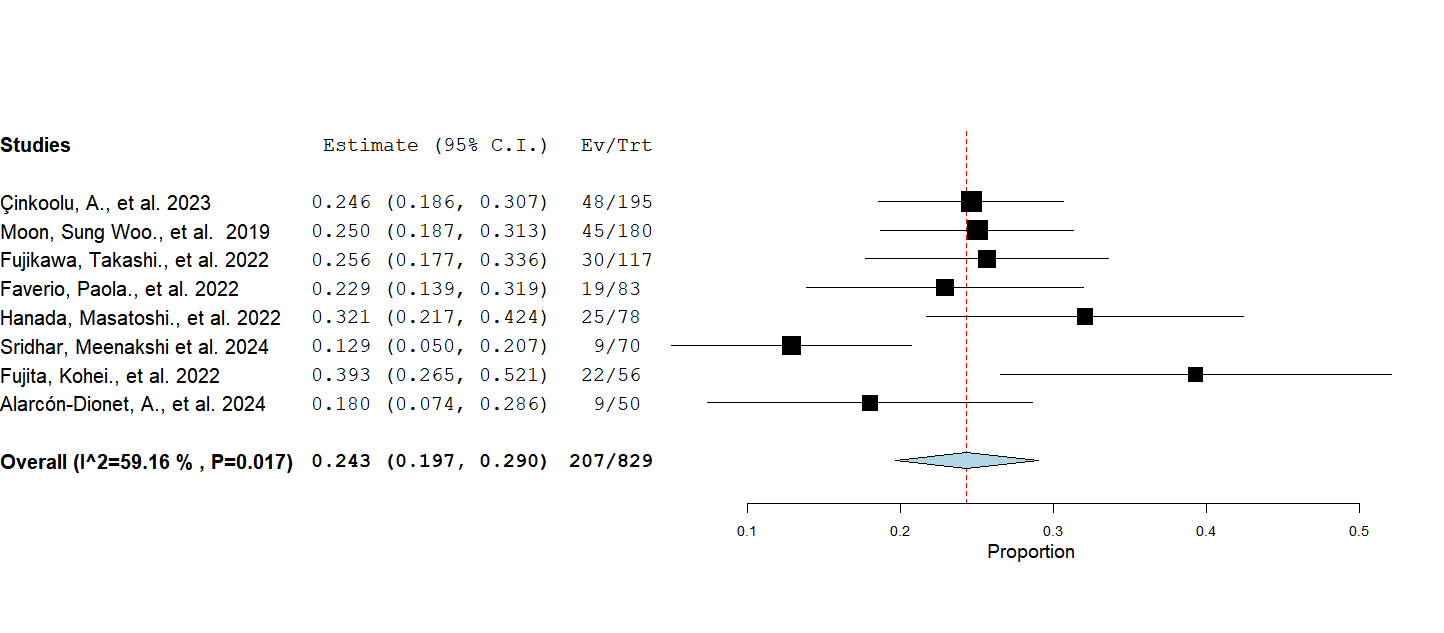


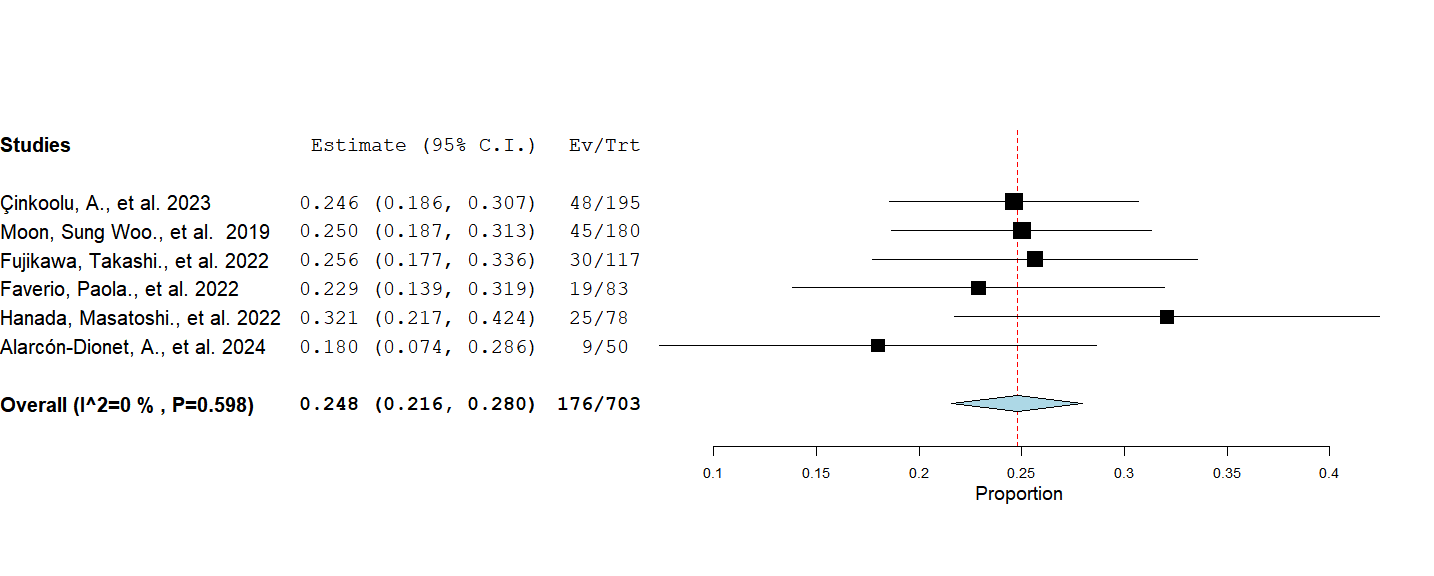


Subgroup prevalence analysis


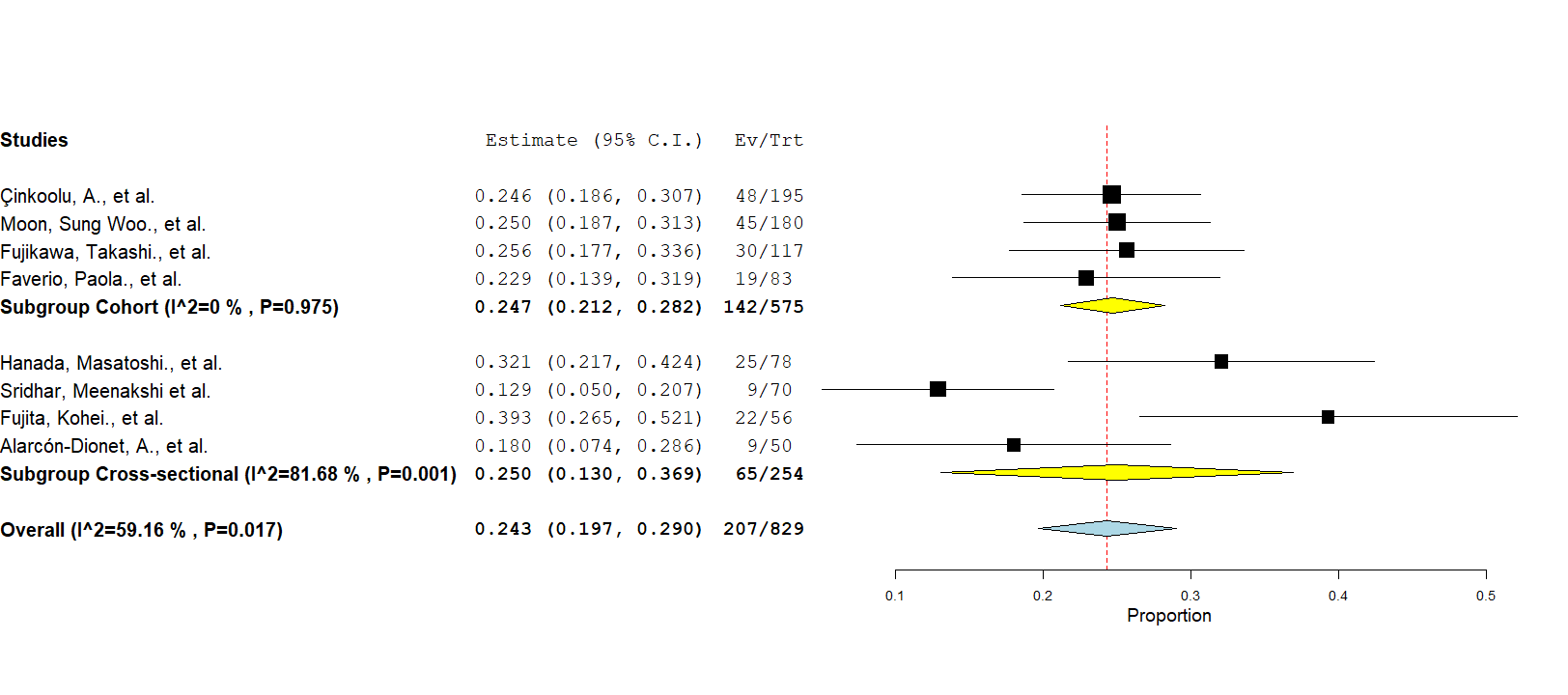


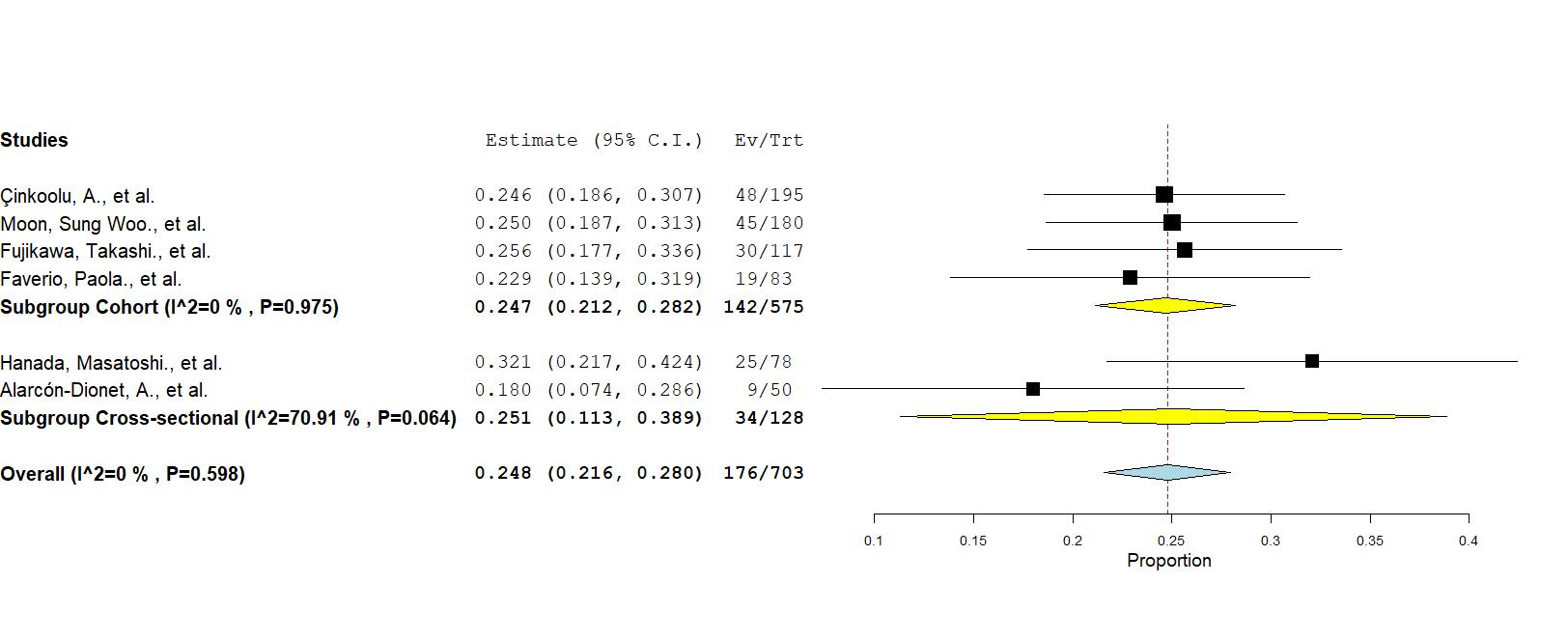


**Sample size**

**
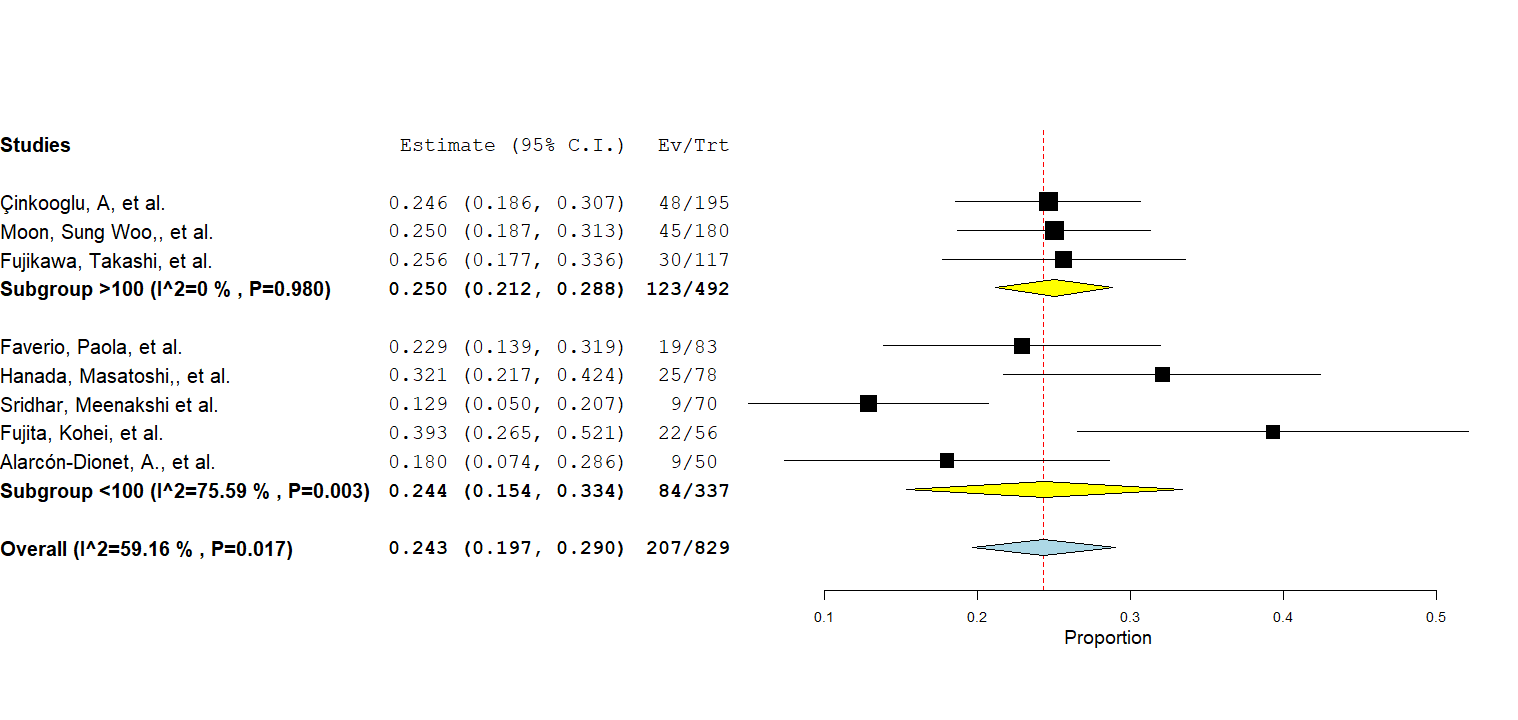
**


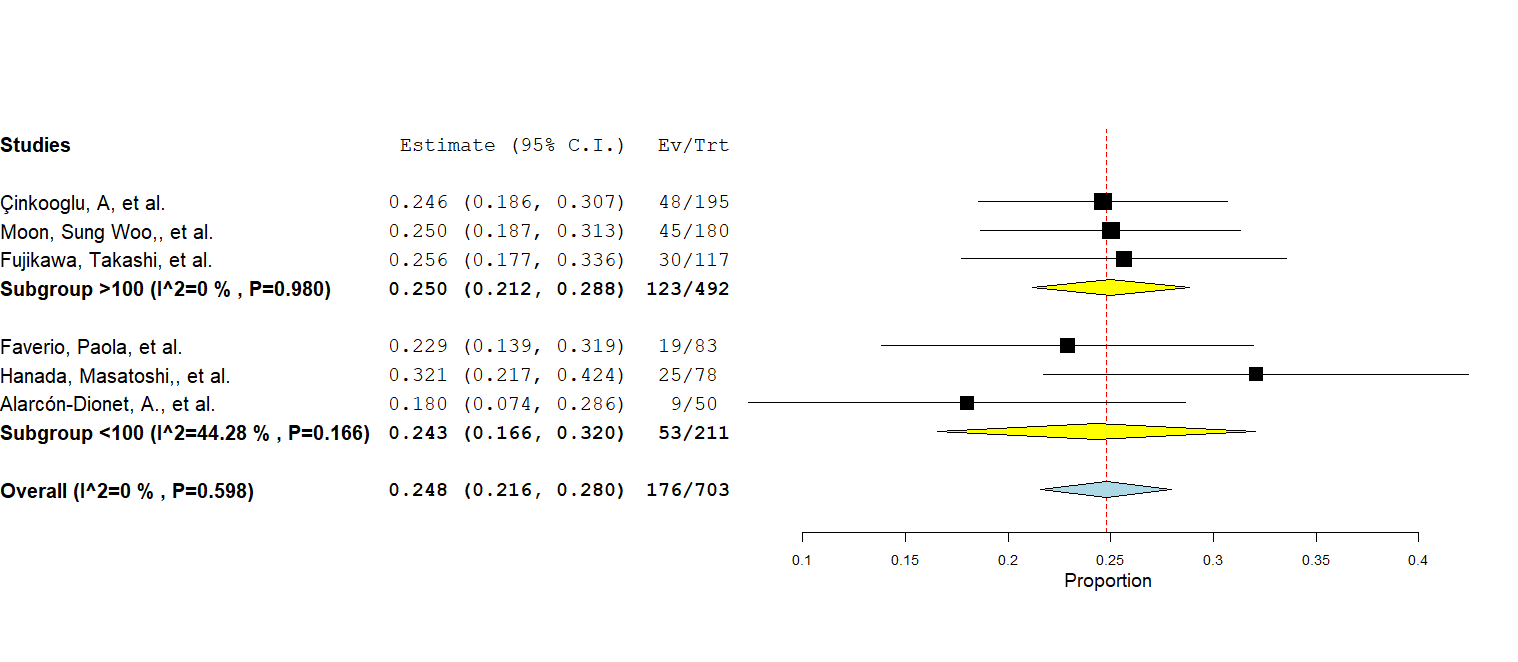


Study Quality


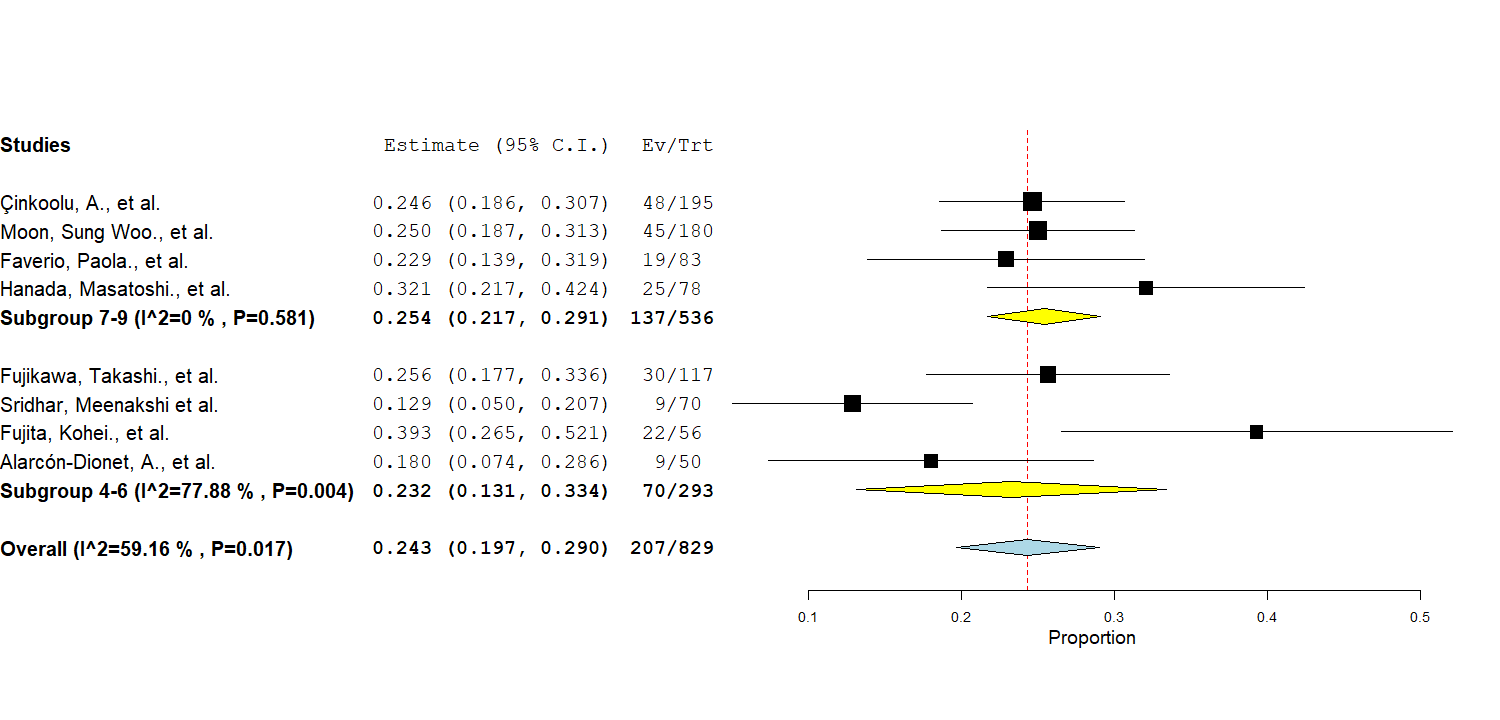


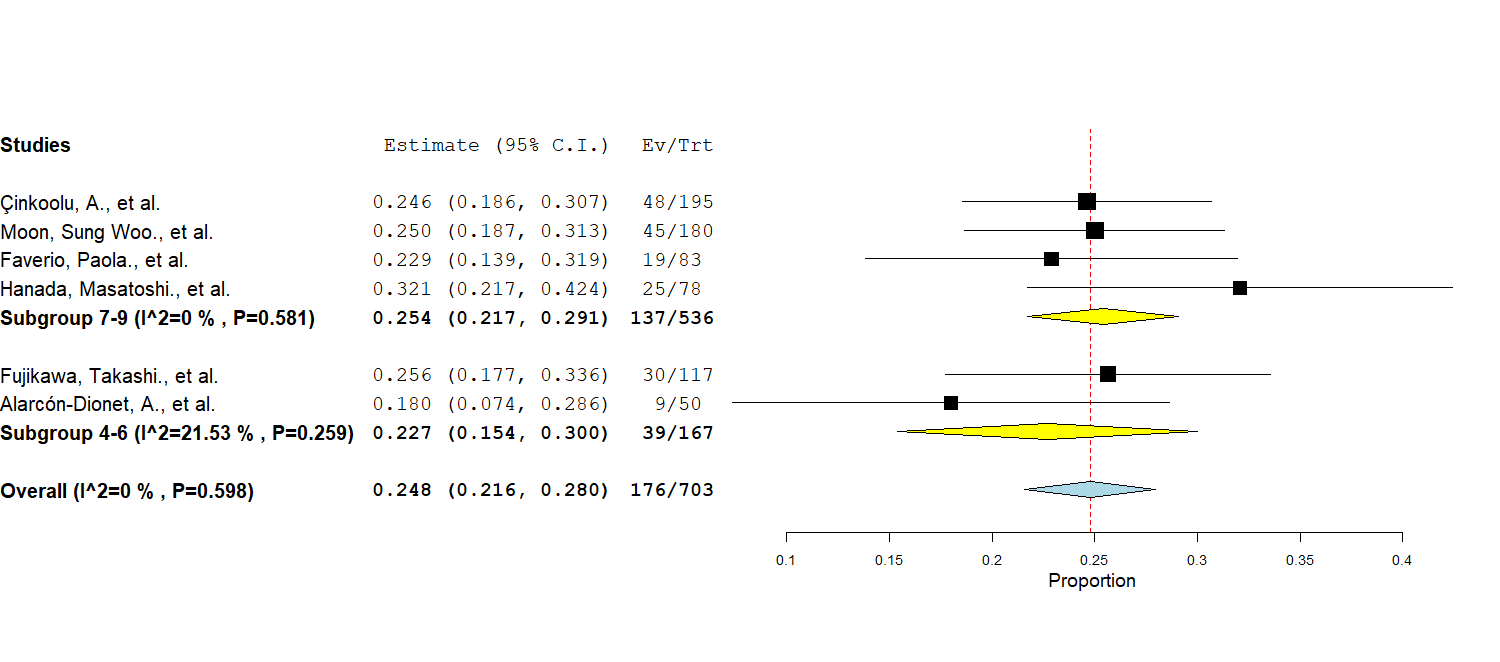


**
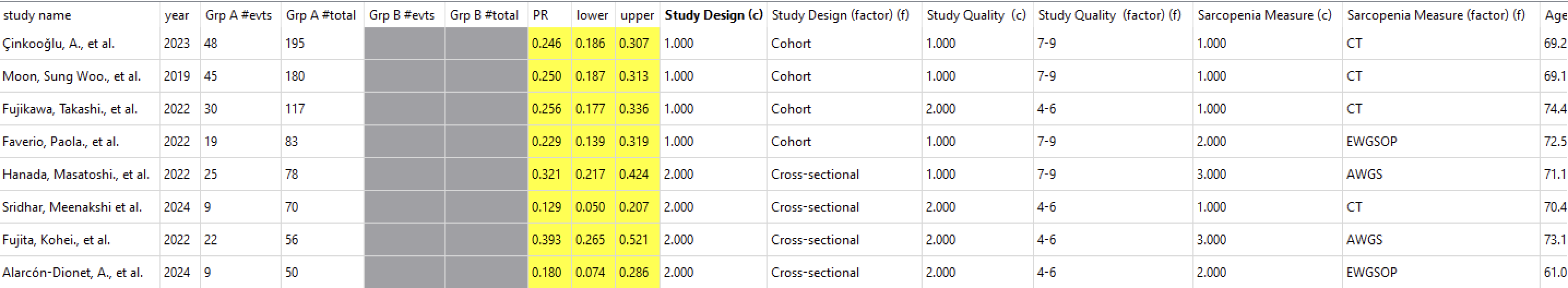
Age**

**Patient Groups**

**
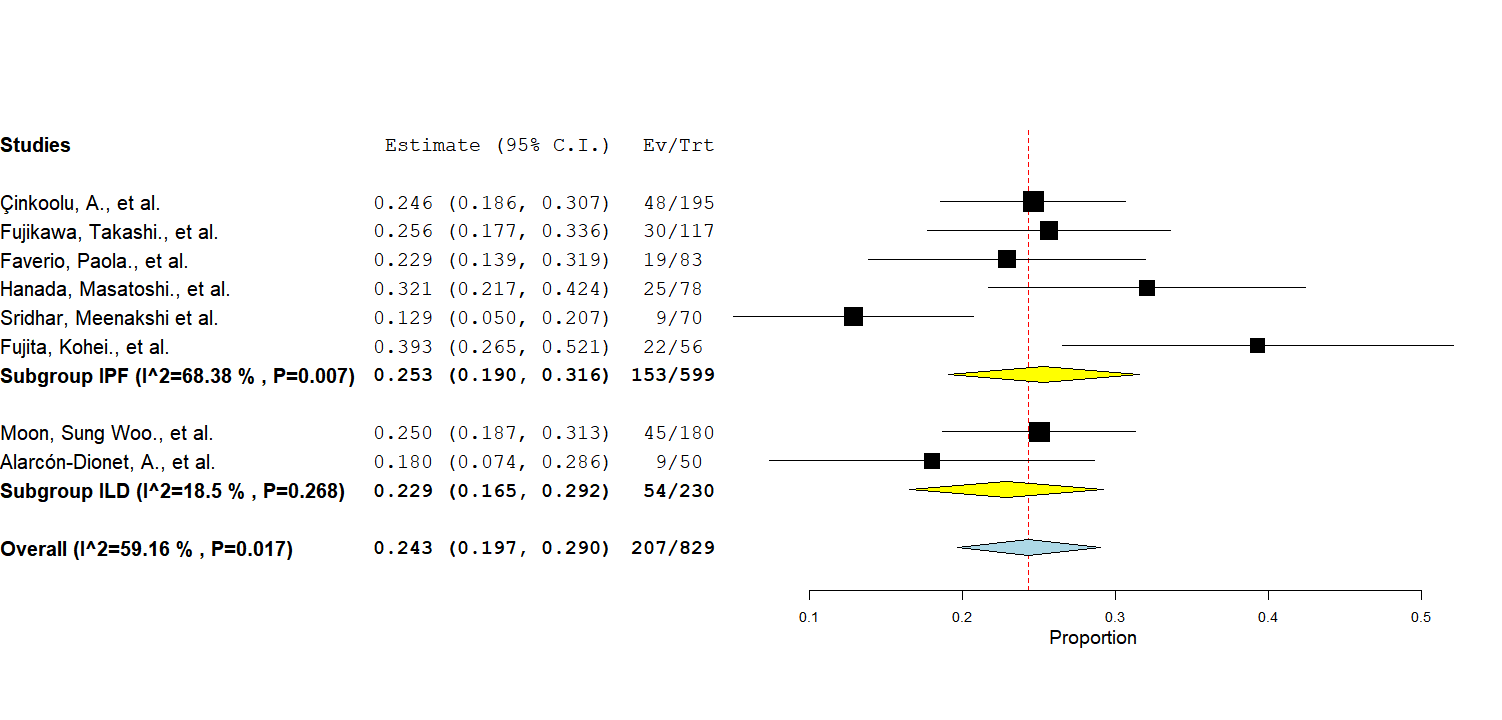
**


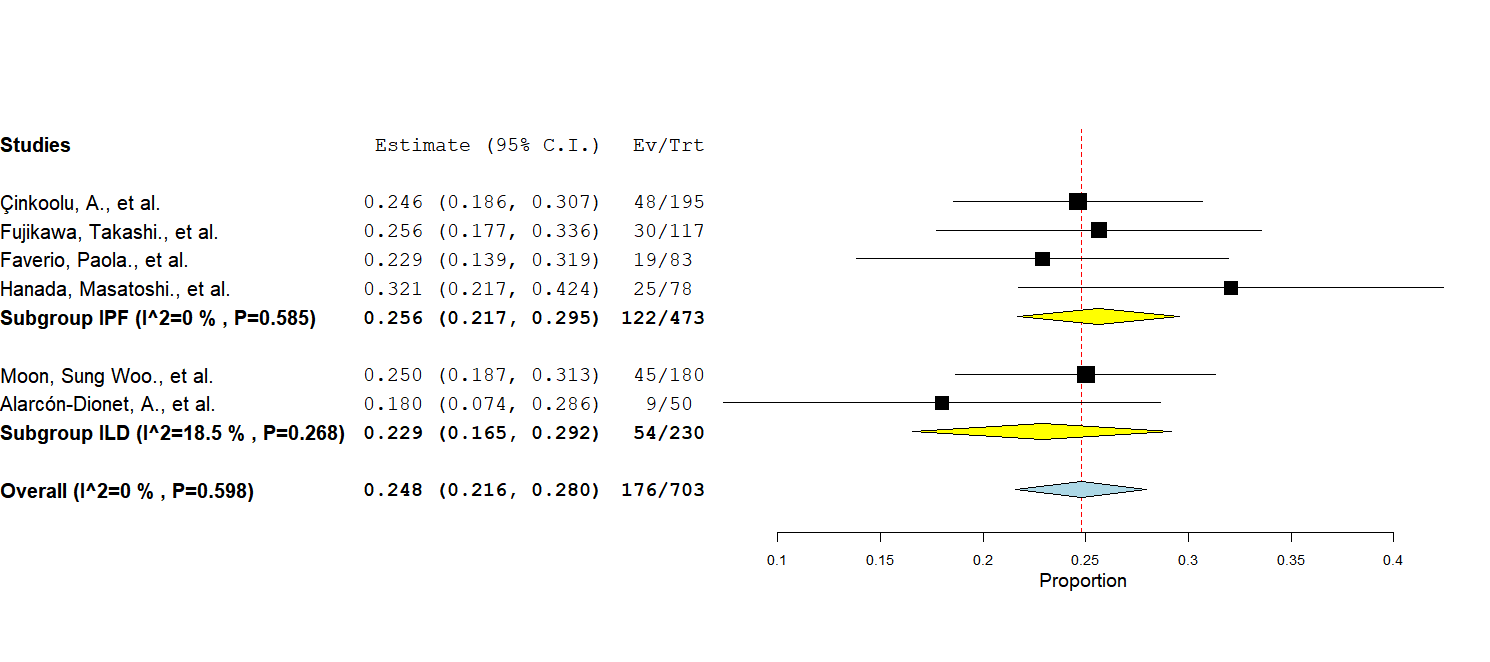


**Sarcopenia Measure**

**
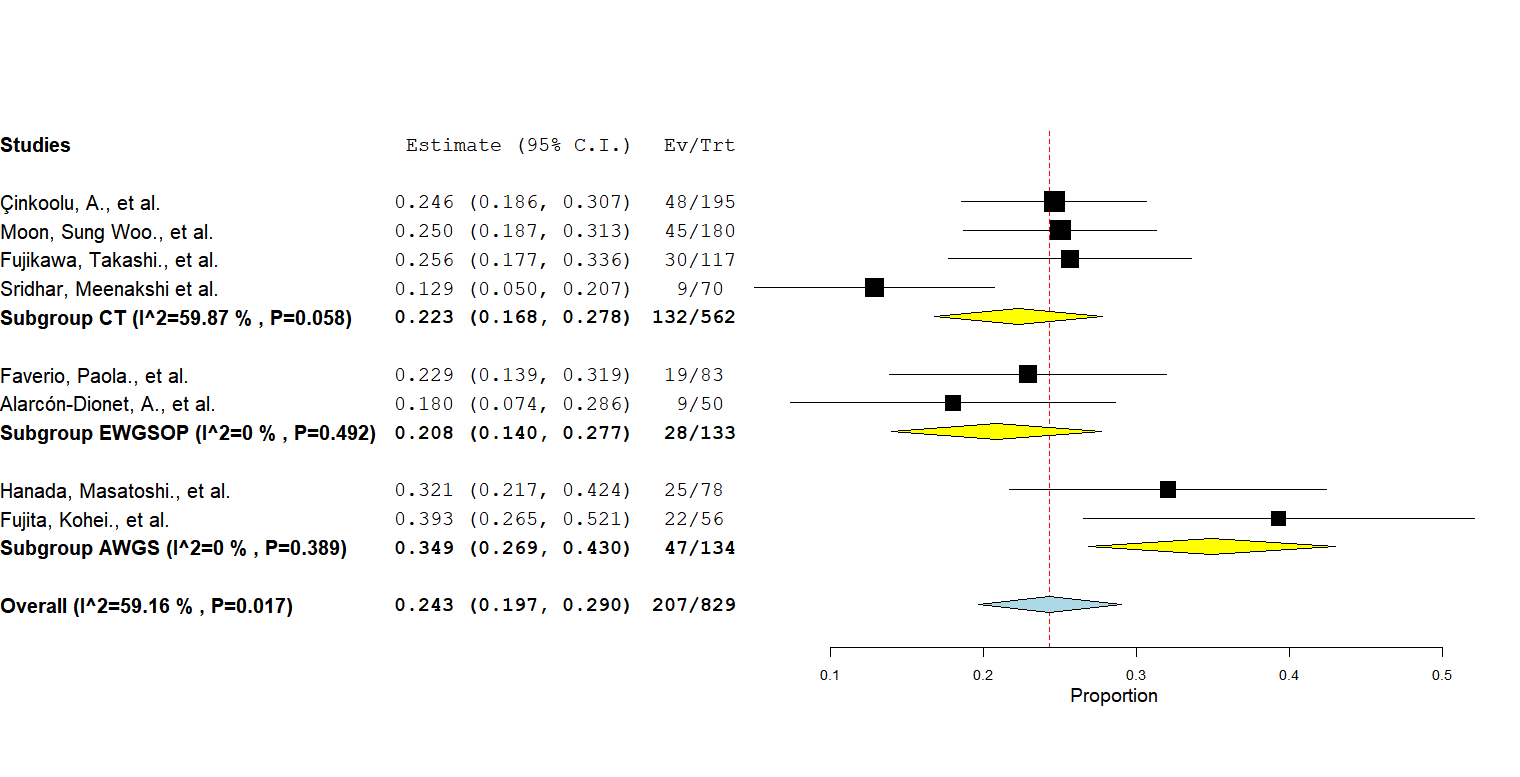
**

**
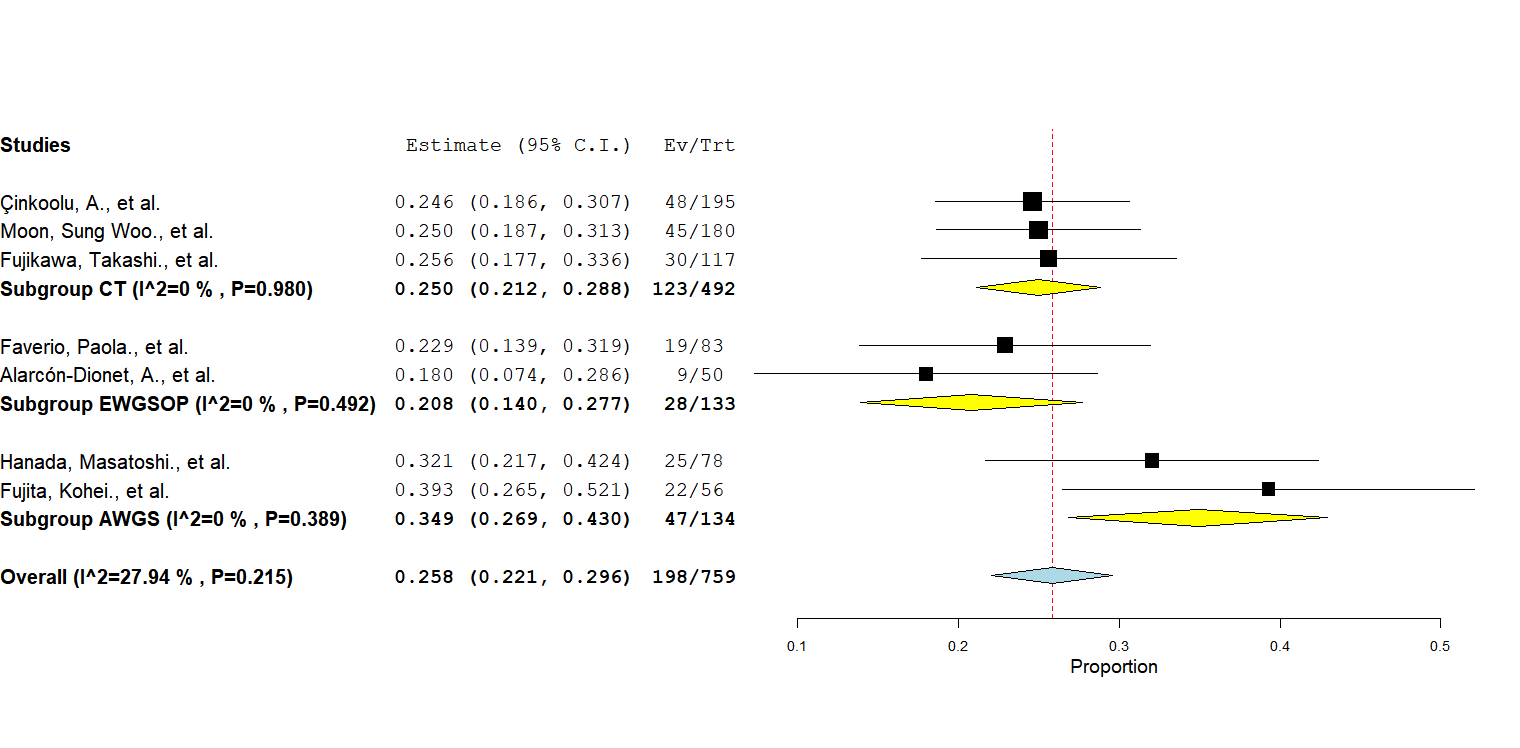
**

**Meta-regression analysis**

**Study Design:**

**
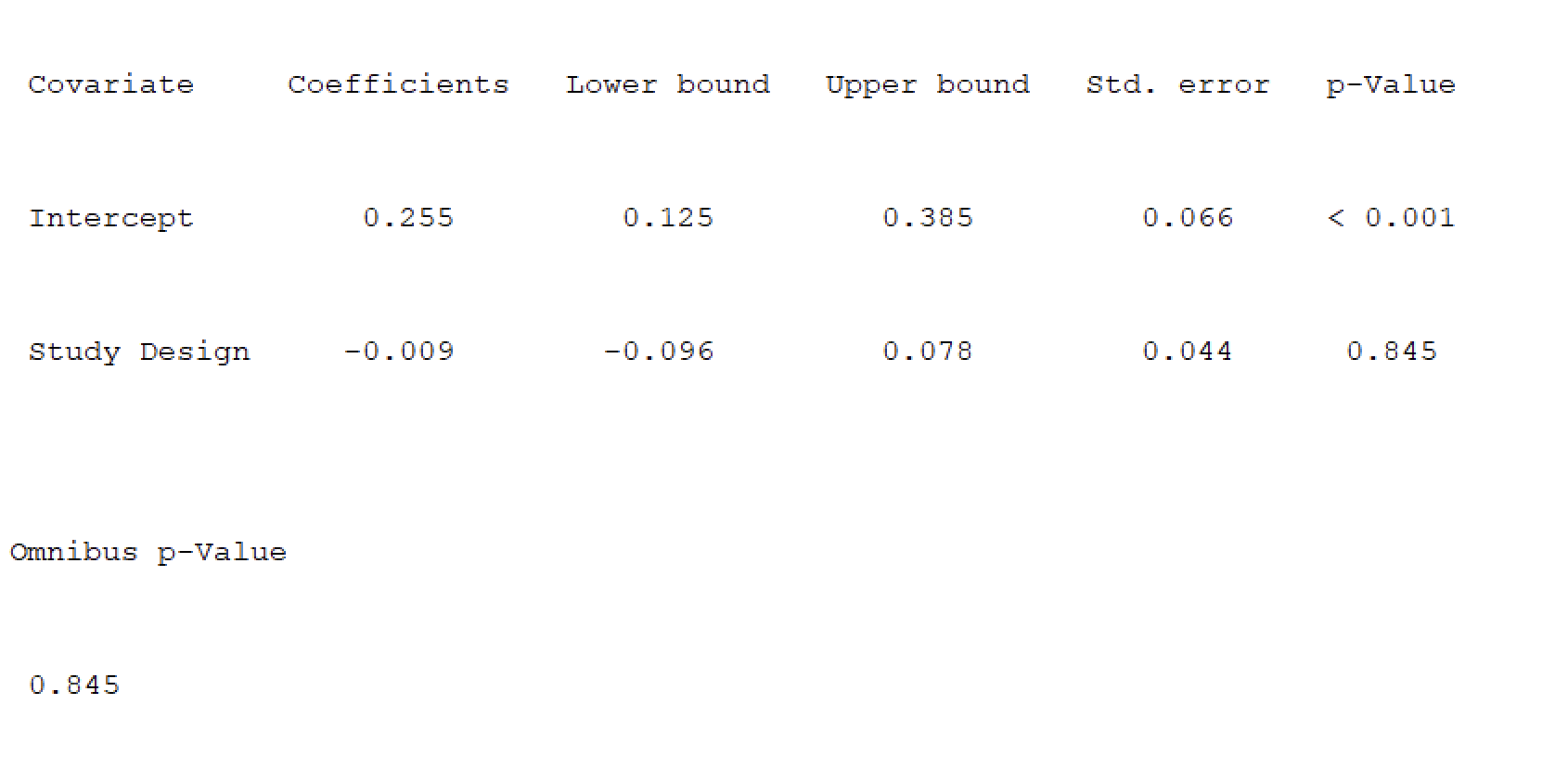
**

**Study quality:**

**
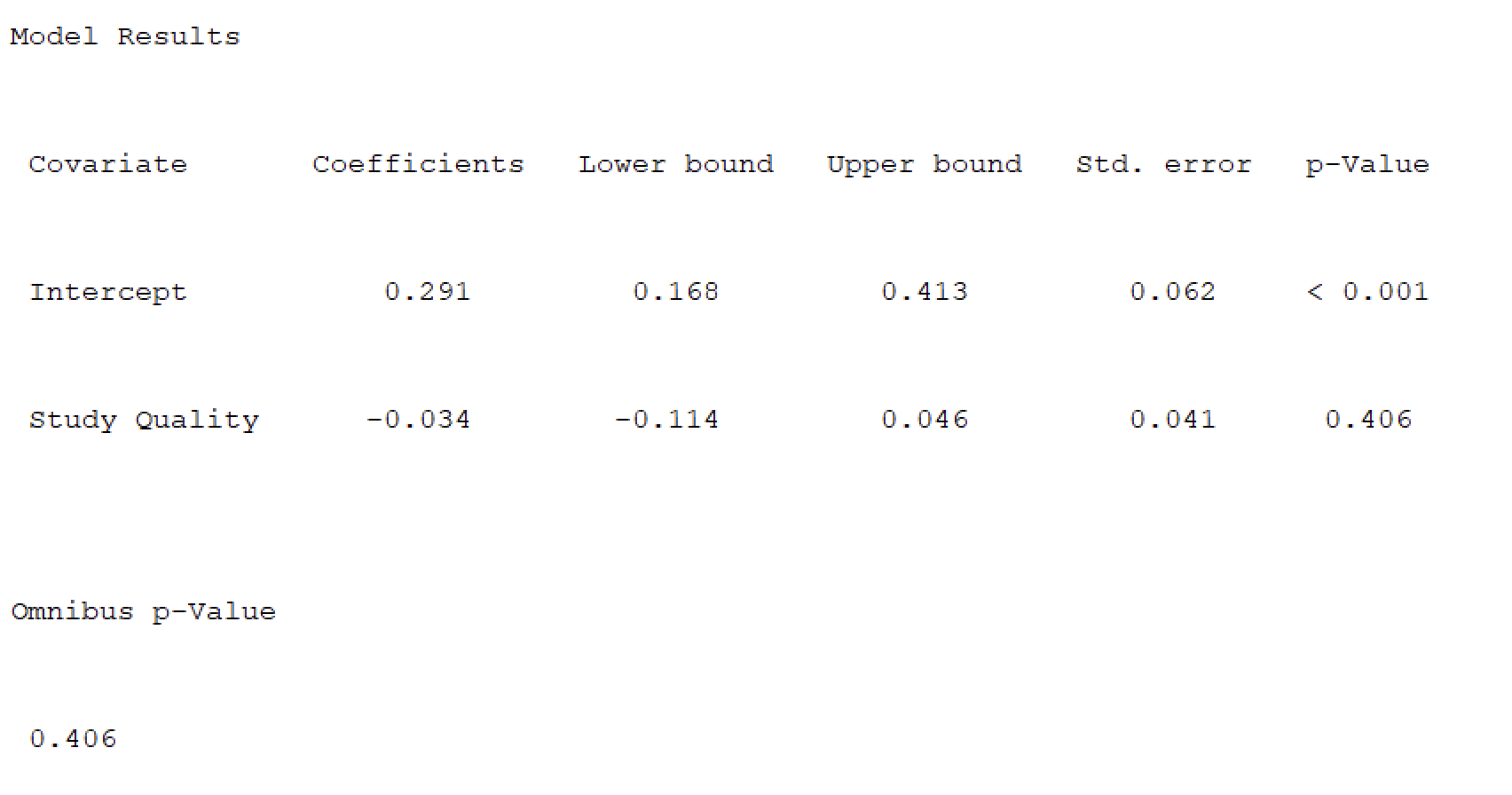
**

**Mean Age:**

**
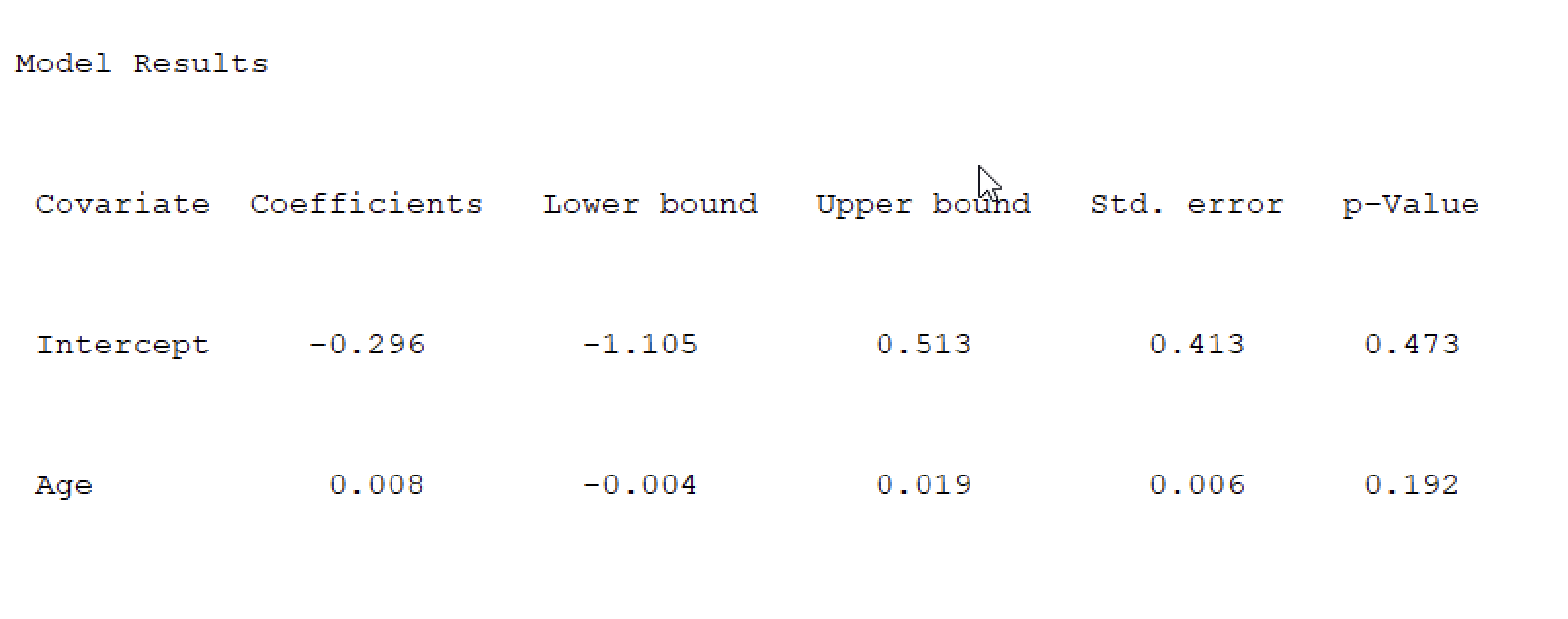
**

**Mean BMI**

**
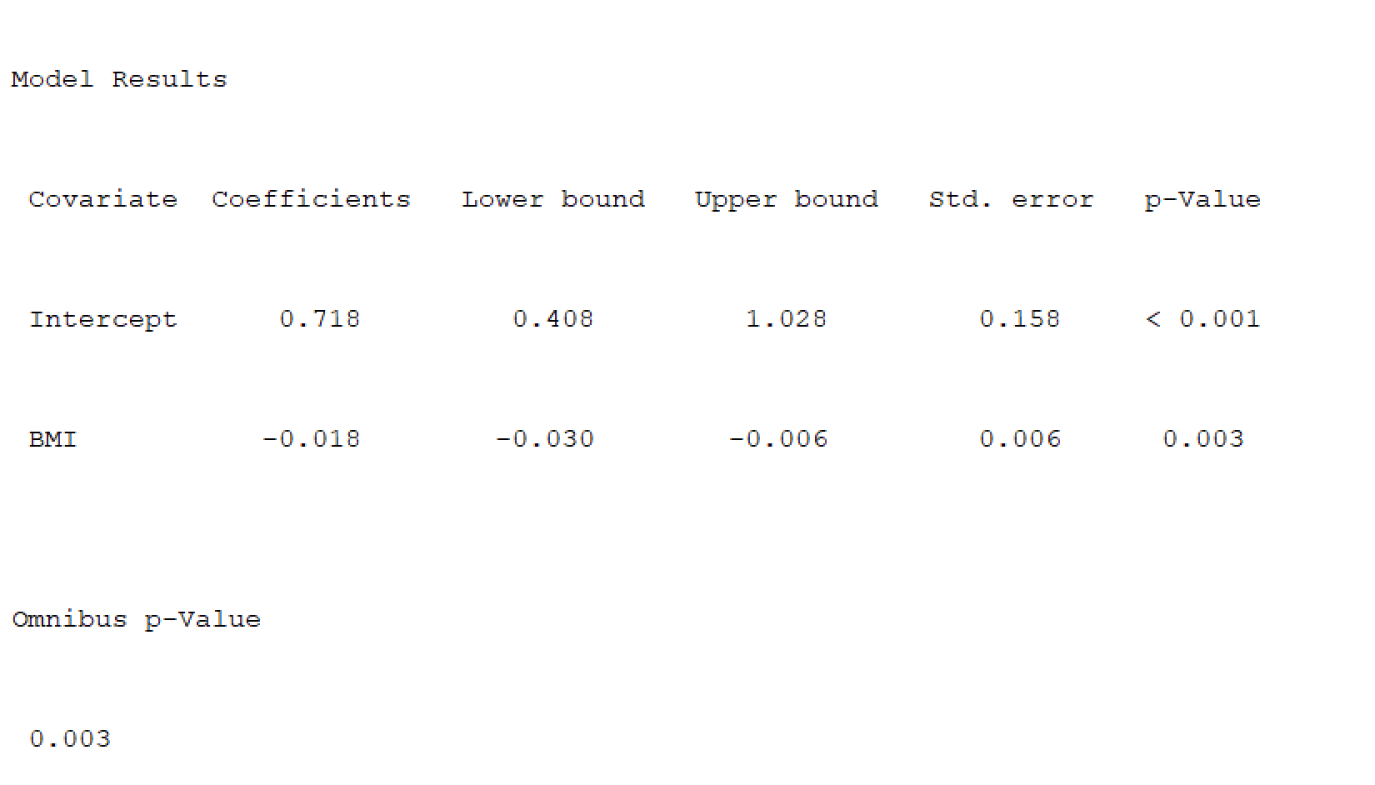
**

**Male percentage**

**
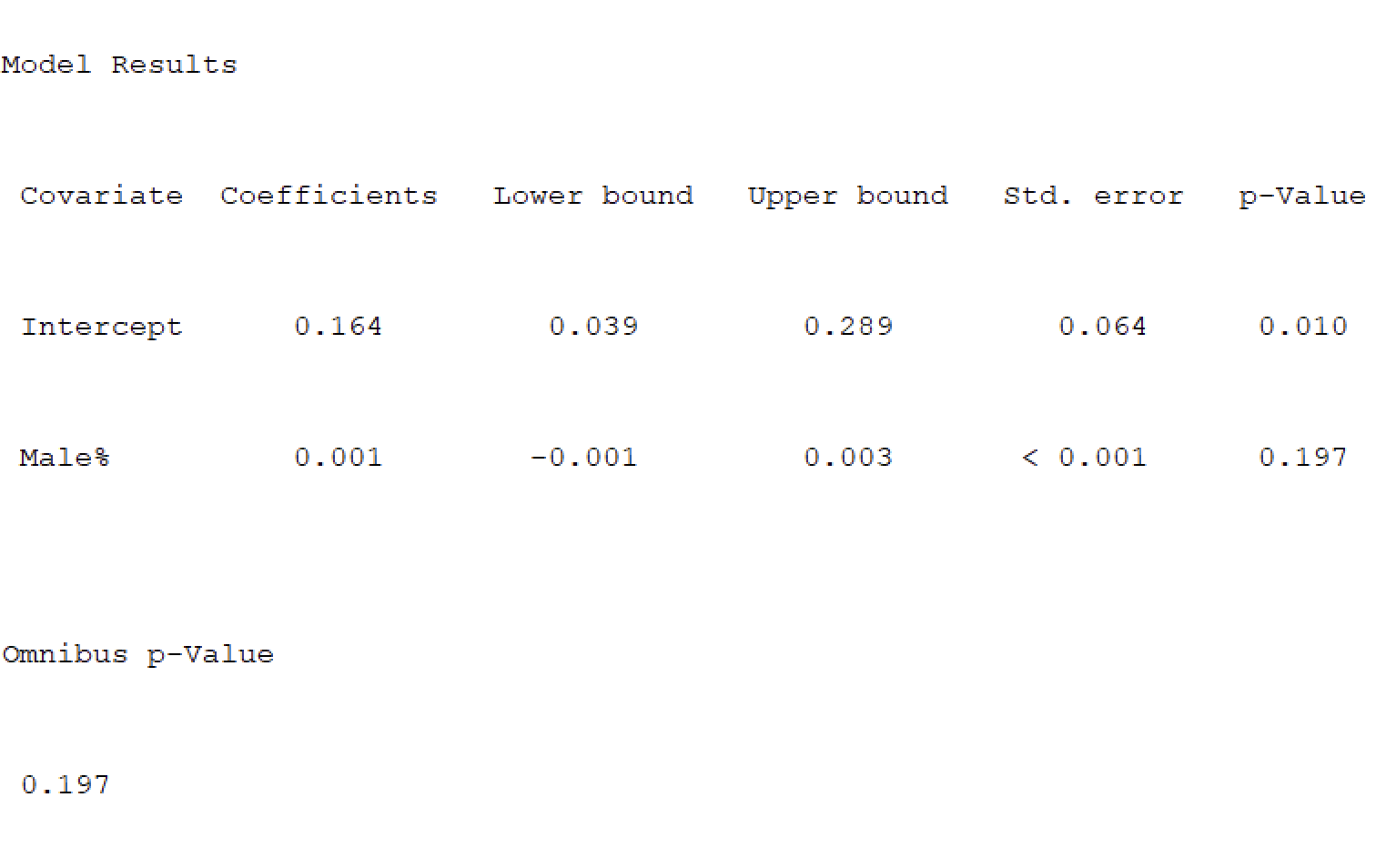
**

**Sarcopenia measure:**

**
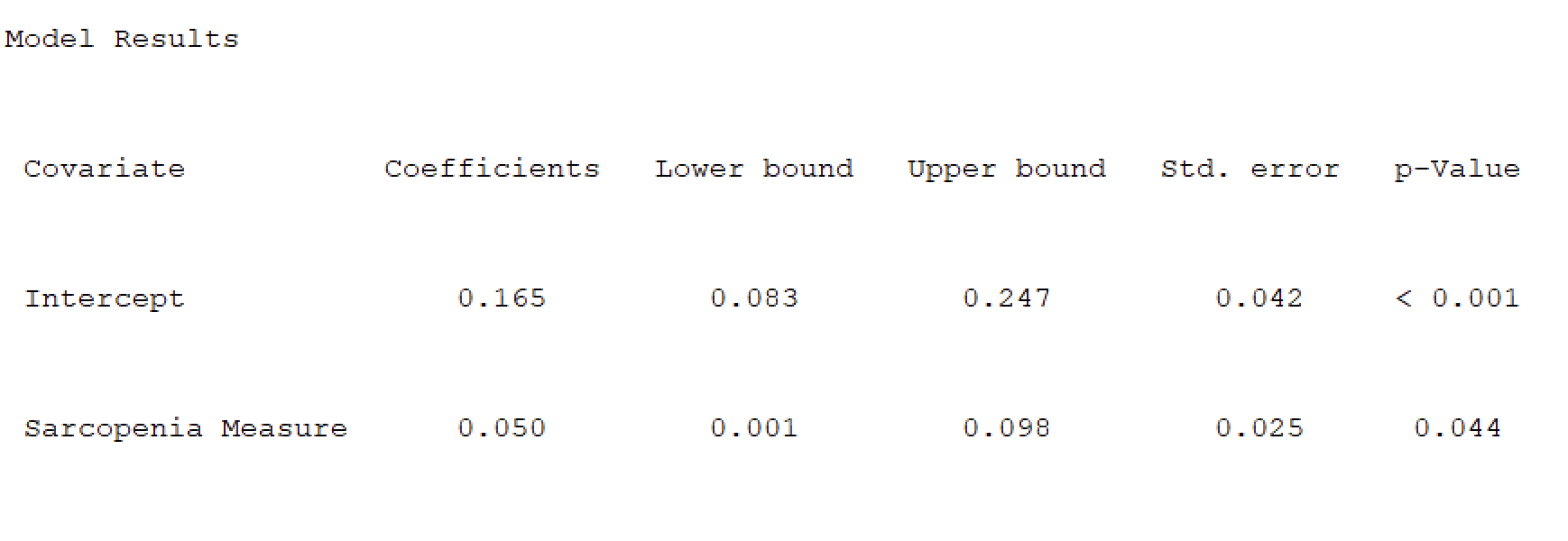
**

**Risk factors data:**

**FVC%**

**
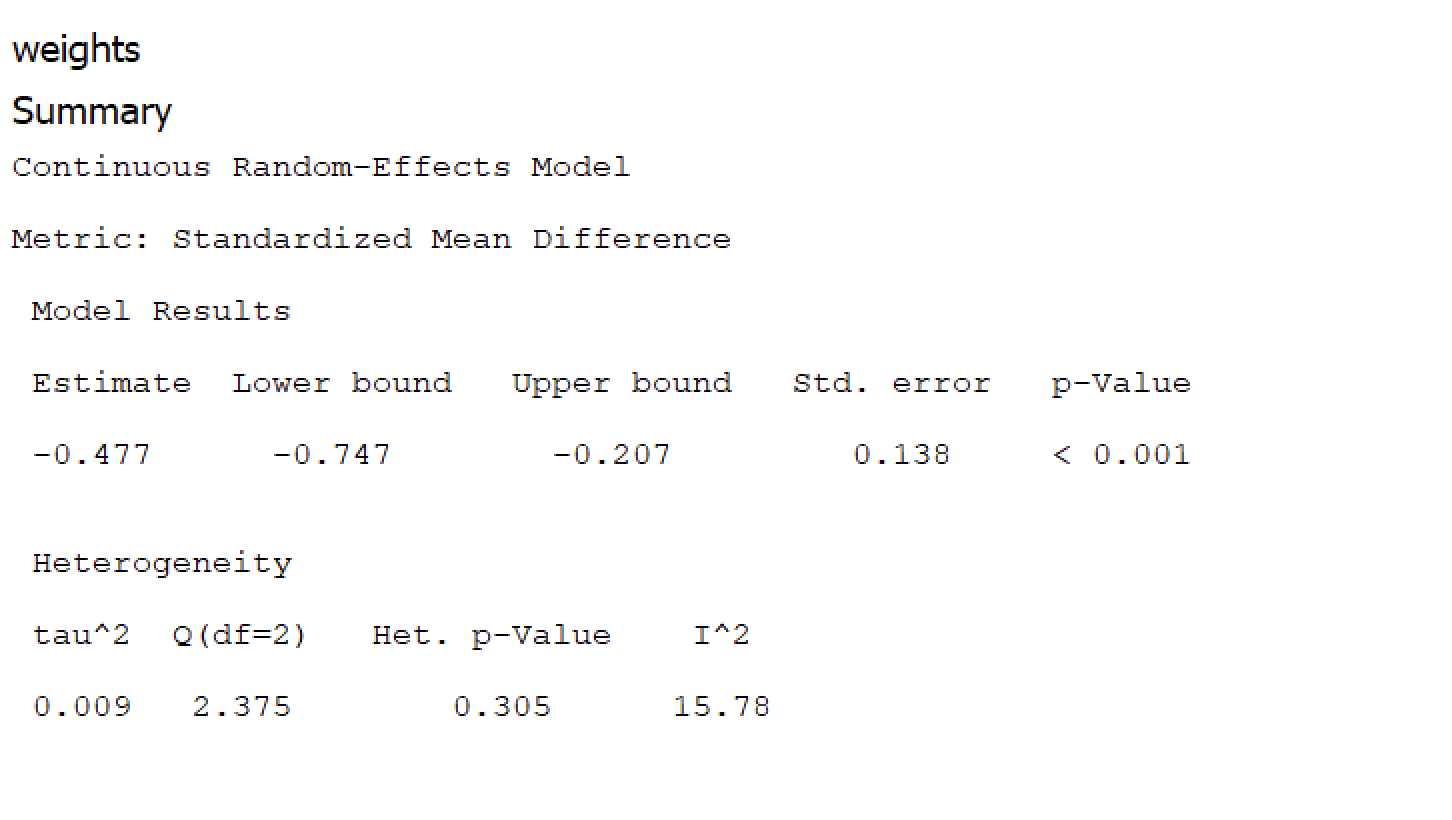
**

**FVC% Predicted**

**
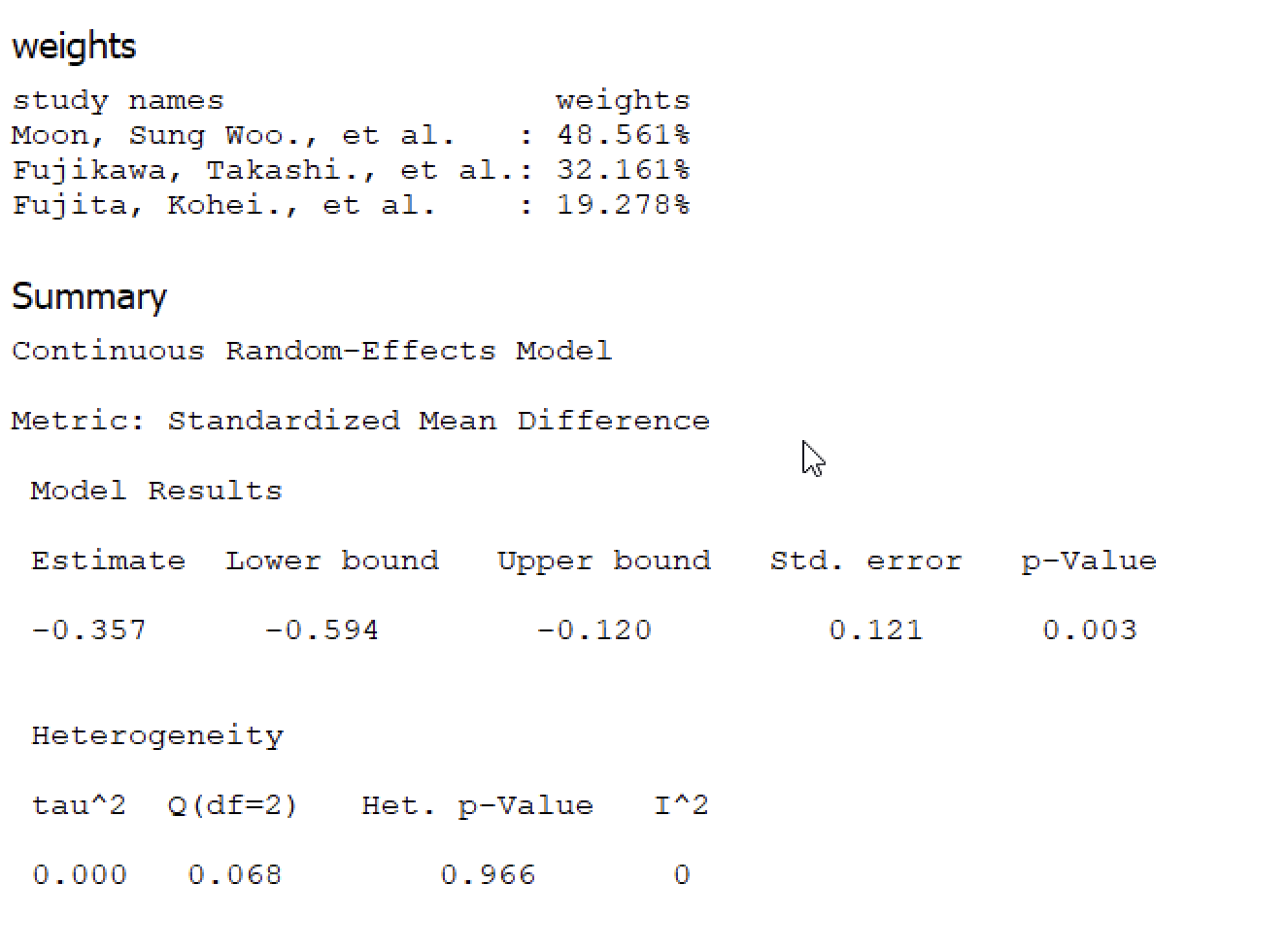
**

**DLCO predicted:**

**
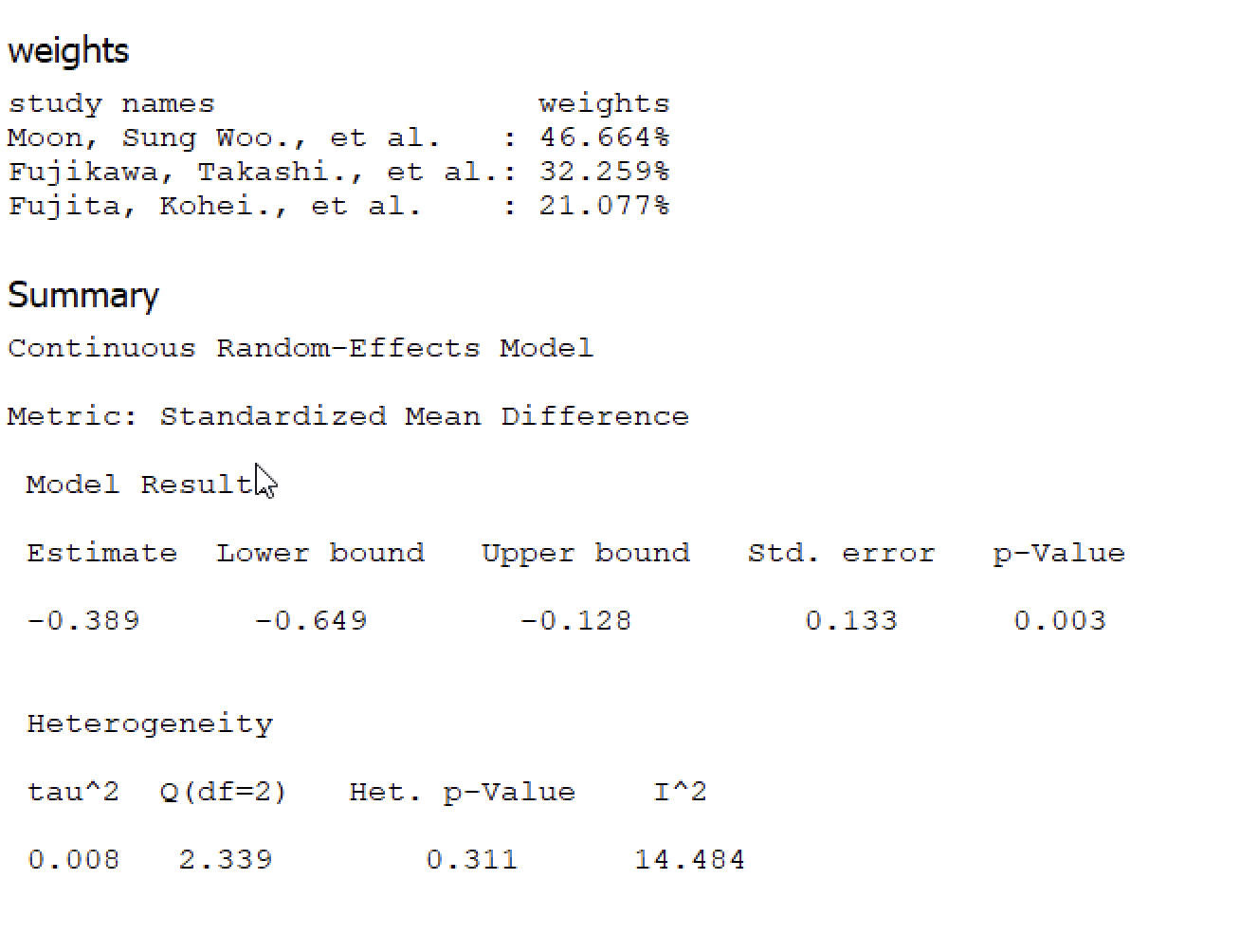
**

**Age:**

**
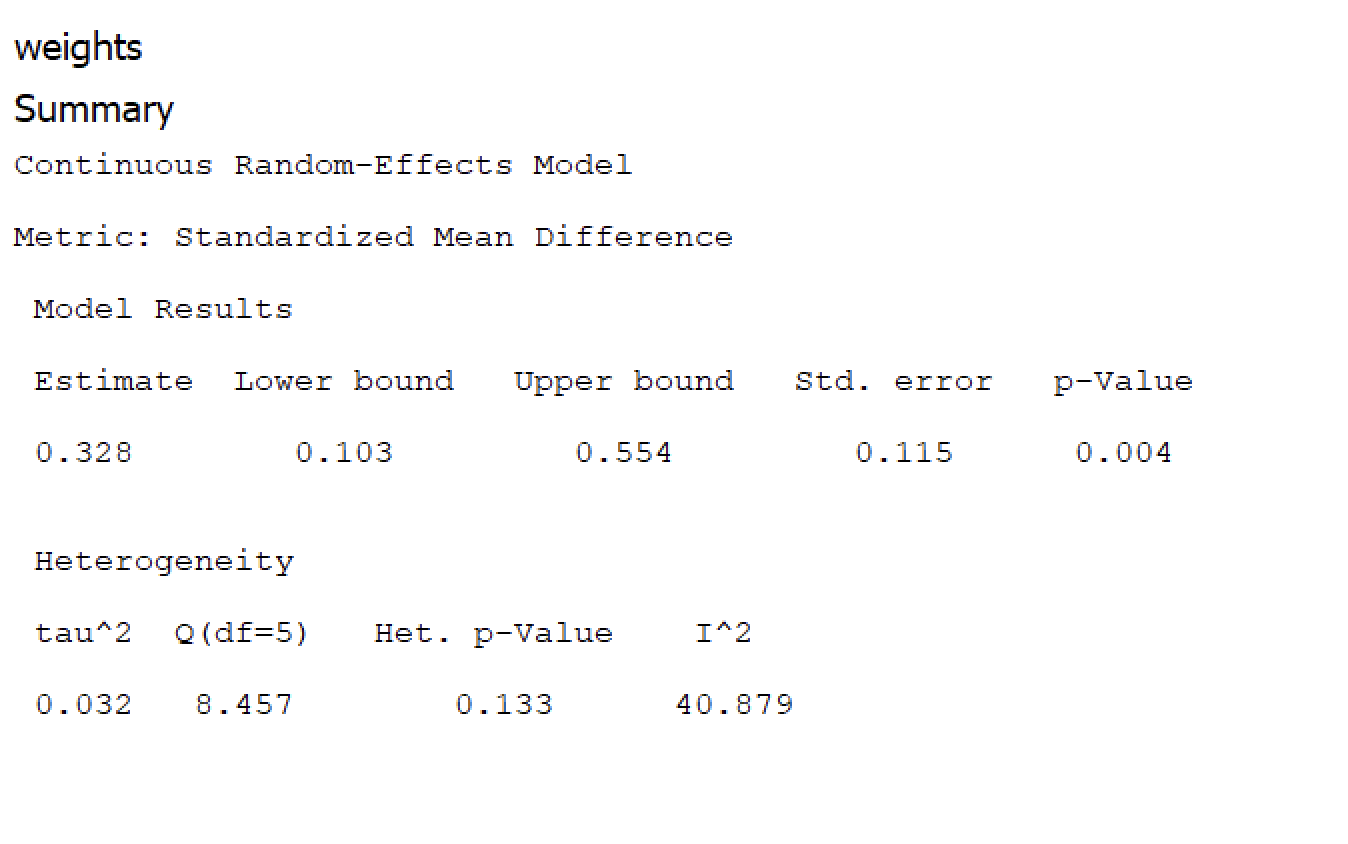
**

**BMI before heterogeneity:**

**
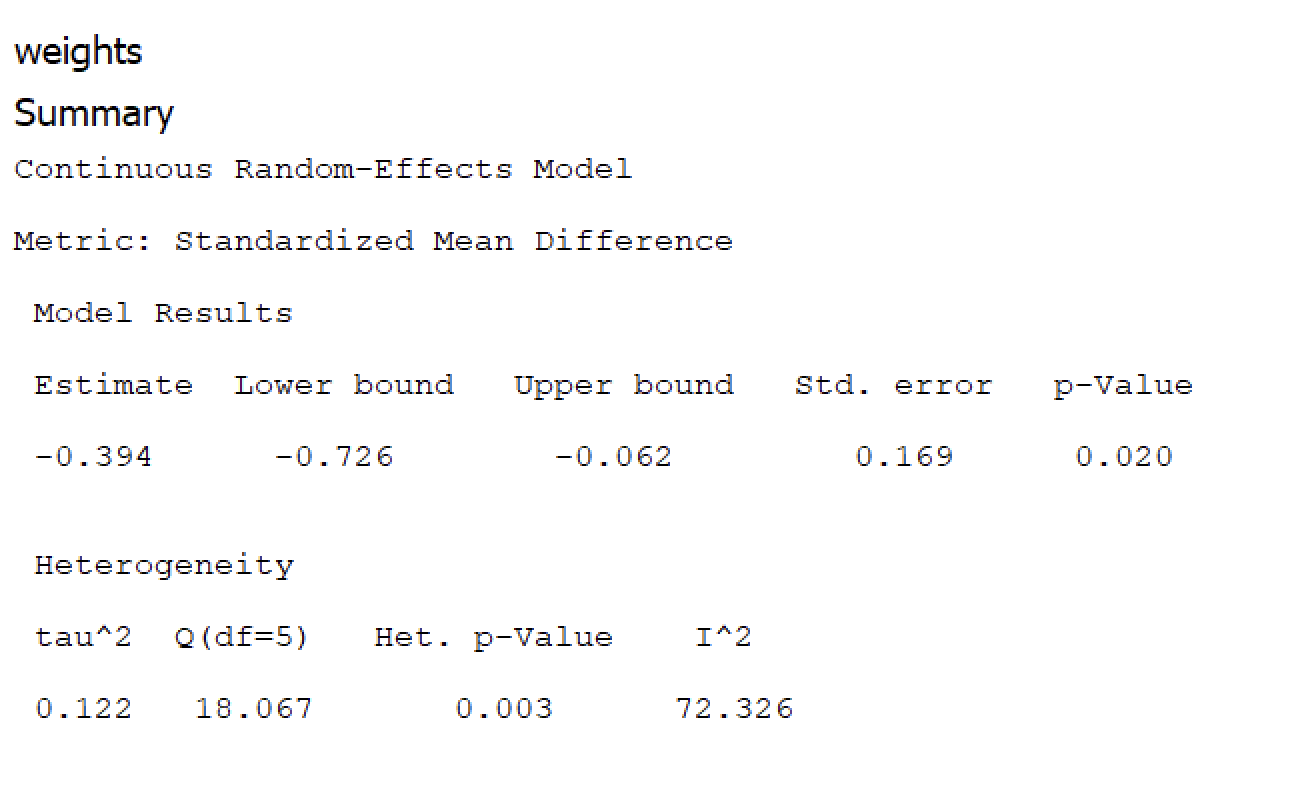
**

**BMI after heterogeneity**

**
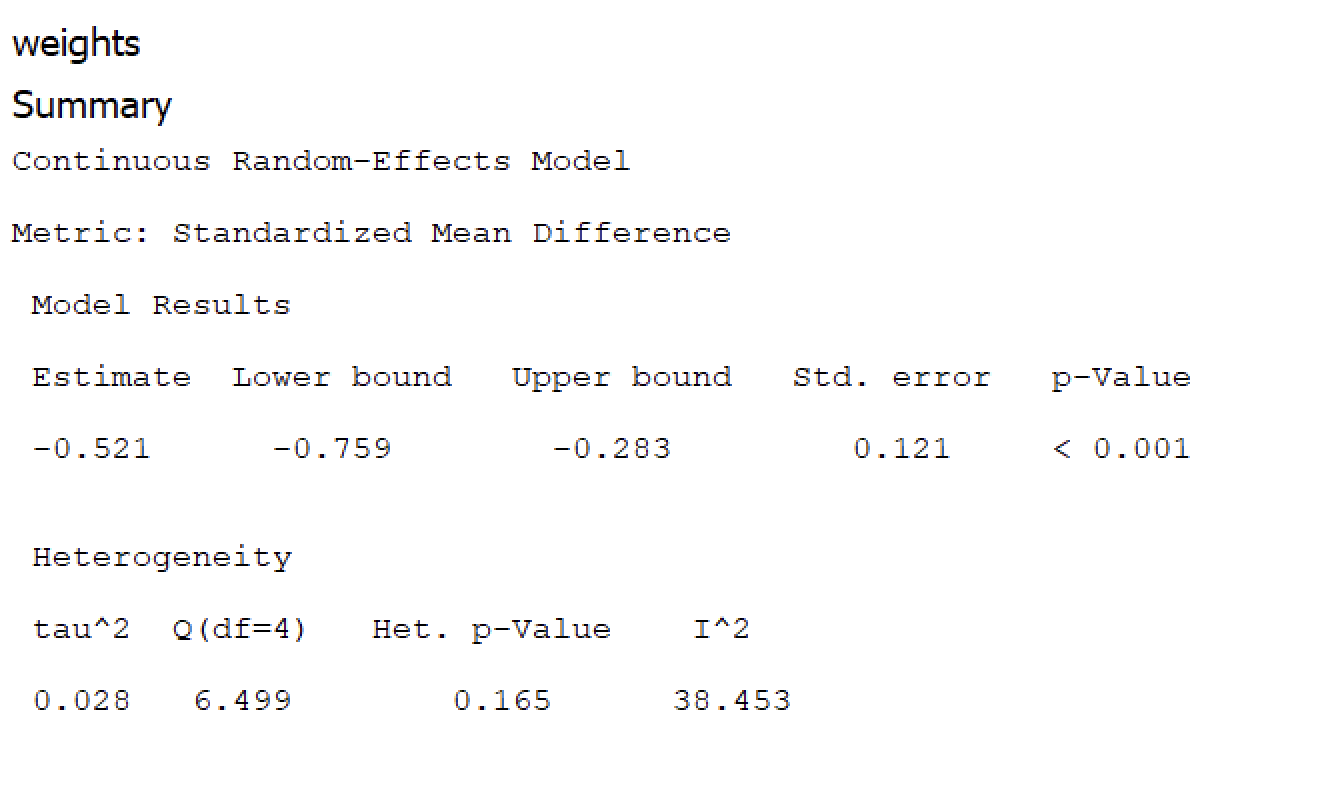
**

**6MWT:**

**
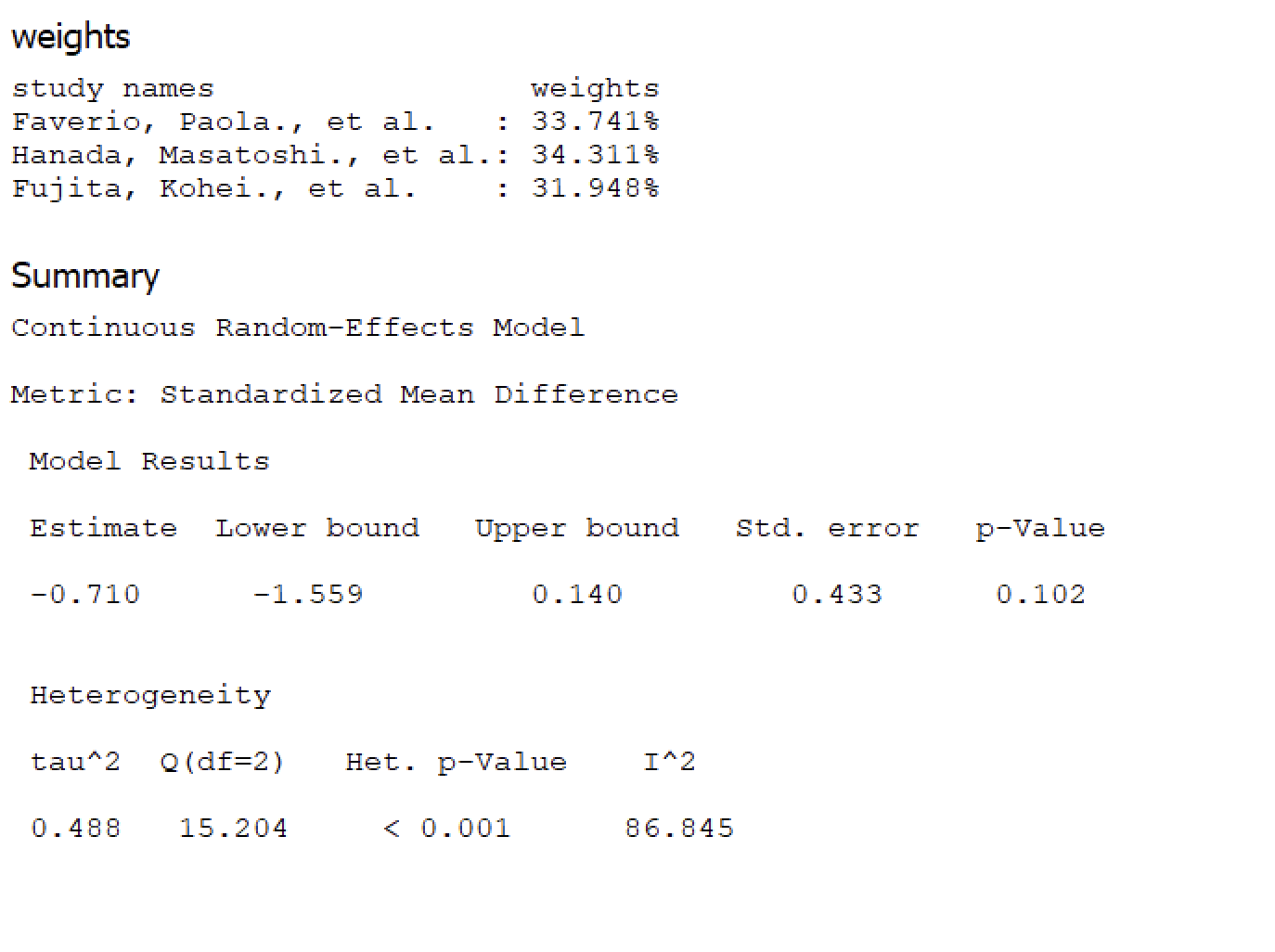
**

**Smoking Risk factors**

**
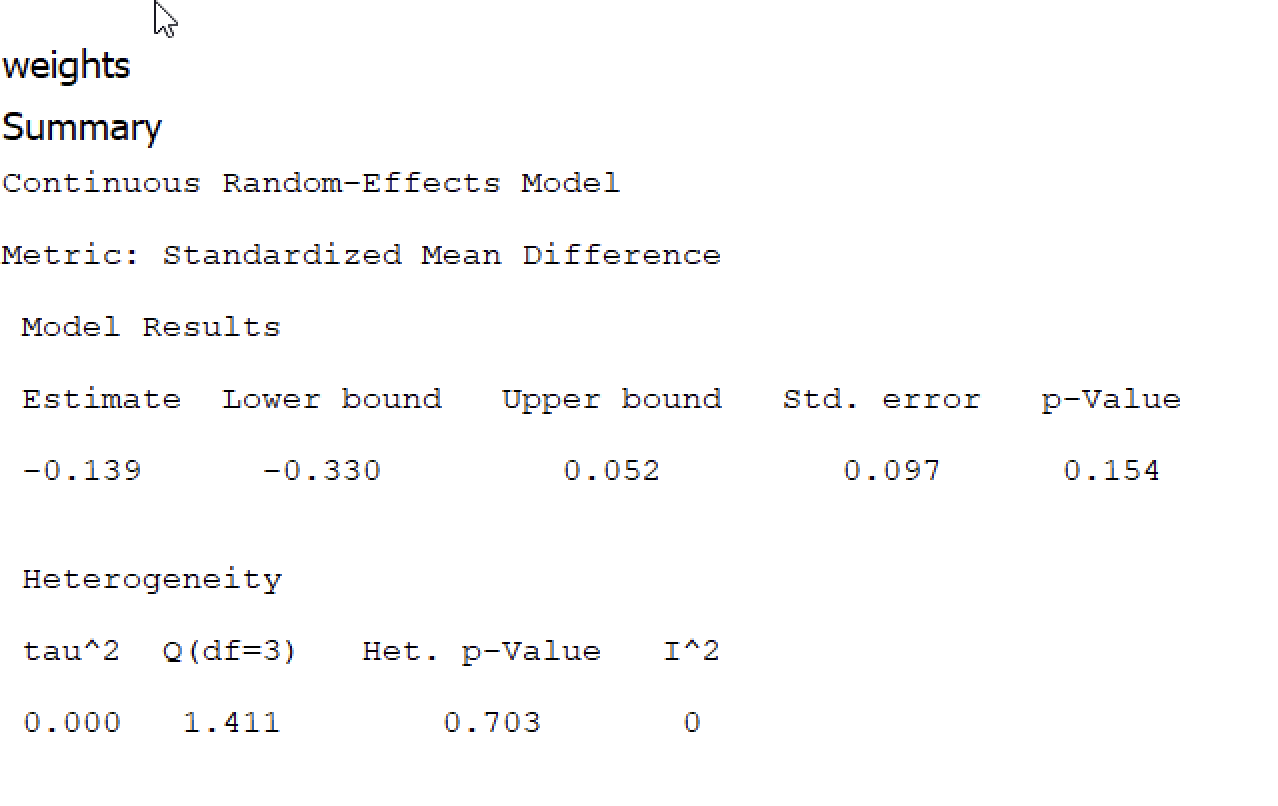
**

**Male %**


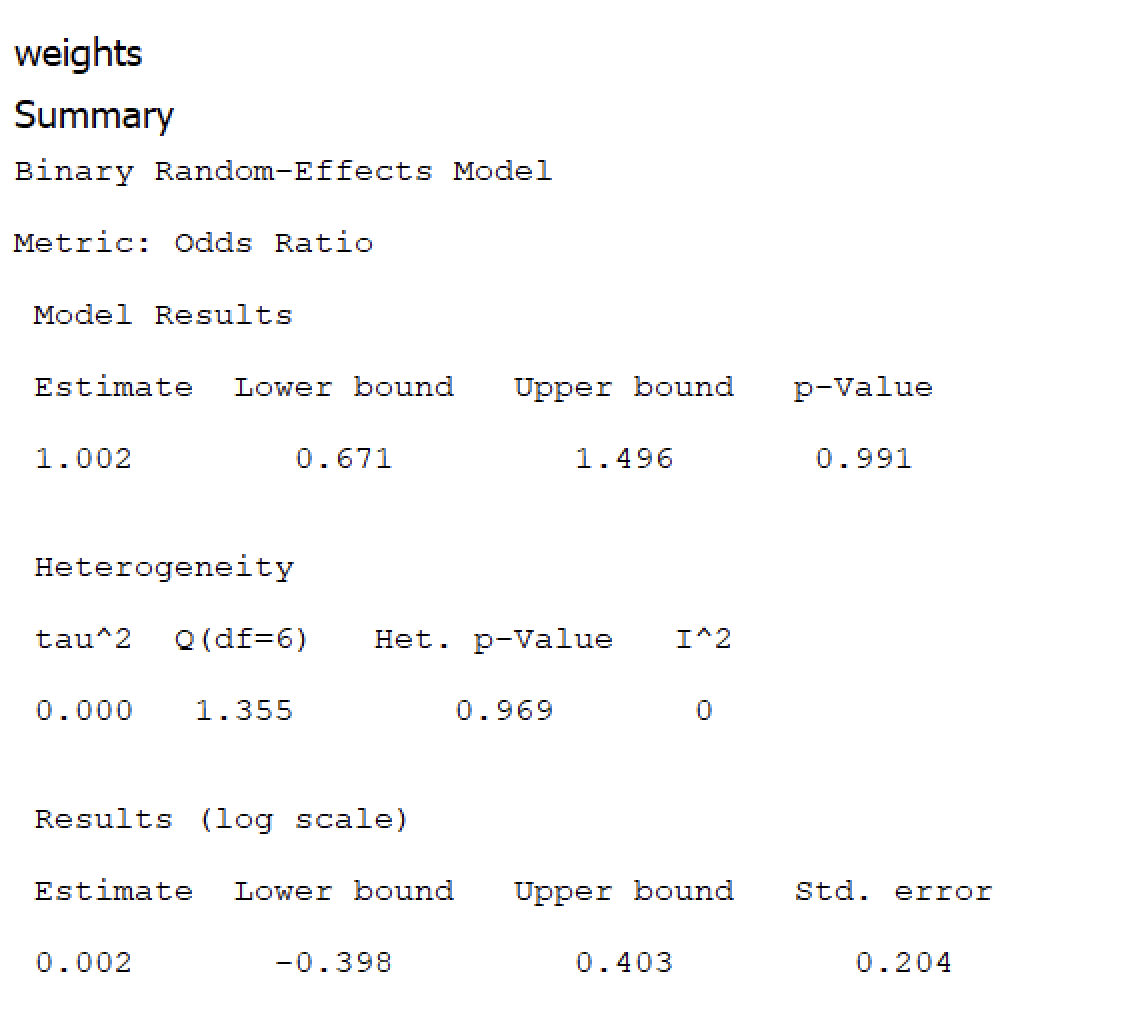


### **Quality Assessment and Publication Bias of the Included Studies**

The quality of the eight included studies (Table 1) were evaluated using the Newcastle-Ottawa Quality Assessment Scale (NOS), a validated tool that assesses non-randomised studies based on selection, comparability, and outcome or exposure domains. Scores ranged from a low of four to a high of nine out of a possible nine. The study by Faverio, Paola., et al^26^. achieved the highest score. Five studies scored between six and seven^23, 25, 27, 28, 30^. The study by Alarcón-Dionet, A., et al^29^. received the lowest score due to several areas where the study did not meet the optimal NOS criteria: selection bias, including only female patients from a single centre, and a limited sample size of 50 patients restricted to only two ILD subtypes. Tests for publication bias and funnel plot asymmetry which would usually be carried out in meta-analyses were not conducted as our meta-analysis included only eight studies^31^.

| **Table 1 \| Newcastle-Ottawa Quality Assessment Scale of the Included Studies** | | | | | | | | | | |
| --- | --- | --- | --- | --- | --- | --- | --- | --- | --- | --- |
| **Newcastle-Ottawa Quality Assessment Scale** | **Selection** | | | | **Comparability** | | **Outcome** | | | **Total Score** |
| **Author** | **Represent Selected**  **Cohort** | **Select non-exposed** | **Ascertain non-exposed** | **Outcome not available at the start** | **Important Aspect** | **Additional Aspect** | **Outcome Assessment** | **Follow up** | **Adequacy of follow-up** |  |
| *Çinkooğlu, A., et al.^25^* | 0 | 1 | 1 | 1 | 1 | 1 | 1 | 1 | 1 | 8 |
| *Moon, Sung Woo., et al.^26^* | 1 | 1 | 1 | 1 | 1 | 1 | 1 | 0 | 0 | 7 |
| *Fujikawa, Takashi., et al.^24^* | 0 | 1 | 1 | 1 | 1 | 1 | 1 | 0 | 0 | 6 |
| *Faverio, Paola., et al.^27^* | 1 | 1 | 1 | 1 | 1 | 1 | 1 | 1 | 1 | 9 |
| *Hanada, Masatoshi., et al.^28^* | 1 | 1 | 1 | 1 | 1 | 1 | 1 | 0 | 0 | 7 |
| *Sridhar, Meenakshi., et al.^31^* | 0 | 1 | 1 | 1 | 0 | 0 | 1 | 1 | 1 | 6 |
| *Fujita, Kohei., et al.^29^* | 0 | 1 | 1 | 1 | 1 | 1 | 1 | 0 | 0 | 6 |
| *Alarcón-Dionet, A., et al.^30^* | 0 | 1 | 1 | 1 | 0 | 0 | 1 | 0 | 0 | 4 |
